# Accuracy of Whole-Genome Prediction Using a Genetic Architecture-Enhanced Variance-Covariance Matrix

**DOI:** 10.1534/g3.114.016261

**Published:** 2015-02-09

**Authors:** Zhe Zhang, Malena Erbe, Jinlong He, Ulrike Ober, Ning Gao, Hao Zhang, Henner Simianer, Jiaqi Li

**Affiliations:** * National Engineering Research Center for Breeding Swine Industry, Guangdong Provincial Key Lab of Agro-animal Genomics and Molecular Breeding, College of Animal Science, South China Agricultural University, Guangzhou 510642, China; † Department of Animal Sciences, Animal Breeding and Genetics Group, Georg-August-Universität Göttingen, Göttingen 37075, Germany

**Keywords:** whole-genome prediction, genetic architecture, trait specific relationship matrix, BLUP|GA, GenPred, shared data resource

## Abstract

Obtaining accurate predictions of unobserved genetic or phenotypic values for complex traits in animal, plant, and human populations is possible through whole-genome prediction (WGP), a combined analysis of genotypic and phenotypic data. Because the underlying genetic architecture of the trait of interest is an important factor affecting model selection, we propose a new strategy, termed BLUP|GA (BLUP-given genetic architecture), which can use genetic architecture information within the dataset at hand rather than from public sources. This is achieved by using a trait-specific covariance matrix (**T**), which is a weighted sum of a genetic architecture part (**S** matrix) and the realized relationship matrix (**G**). The algorithm of BLUP|GA (BLUP-given genetic architecture) is provided and illustrated with real and simulated datasets. Predictive ability of BLUP|GA was validated with three model traits in a dairy cattle dataset and 11 traits in three public datasets with a variety of genetic architectures and compared with GBLUP and other approaches. Results show that BLUP|GA outperformed GBLUP in 20 of 21 scenarios in the dairy cattle dataset and outperformed GBLUP, BayesA, and BayesB in 12 of 13 traits in the analyzed public datasets. Further analyses showed that the difference of accuracies for BLUP|GA and GBLUP significantly correlate with the distance between the **T** and **G** matrices. The new strategy applied in BLUP|GA is a favorable and flexible alternative to the standard GBLUP model, allowing to account for the genetic architecture of the quantitative trait under consideration when necessary. This feature is mainly due to the increased similarity between the trait-specific relationship matrix (**T** matrix) and the genetic relationship matrix at unobserved causal loci. Applying BLUP|GA in WGP would ease the burden of model selection.

With the availability of high-density single-nucleotide polymorphisms (SNPs) covering the whole genome of many animal and plant species, the genetic merit of a genotyped individual can be more accurately estimated via a combined analysis of phenotypes and genotypes. This method usually is termed genomic prediction (Meuwissen *et al.* 2001) in animal and plant breeding and is the cornerstone of genomic selection programs (Goddard and Hayes 2009). Equivalently, whole-genome prediction (WGP) (de los Campos *et al.* 2010) in human genetics aims at an improved prediction of complex disease phenotypes based on genome-wide markers. Recently, WGP was paid close attention by scientists from animal breeding (Goddard and Hayes 2009; Wolc *et al.* 2011; Cleveland *et al.* 2012), plant breeding (Resende *et al.* 2012; Riedelsheimer *et al.* 2012a; Guo *et al.* 2013), and human genetics (Vazquez *et al.* 2012; de los Campos *et al.* 2013b; Dudbridge 2013). In many countries, WGP has been implemented successfully in dairy cattle breeding programs as the standard genetic evaluation method because of its potentiality in accelerating genetic progress (Vanraden *et al.* 2009; Lund *et al.* 2011; Ding *et al.* 2013). The genotypic data analyzed in WGP are not only from high-density SNP chips but also from whole-genome sequencing (Meuwissen and Goddard 2010; Ober *et al.* 2012).

A critical concern in WGP is its predictive ability, which is usually measured as the correlation between the predicted genetic values and the true genetic/phenotypic values in a validation population (Meuwissen *et al.* 2001; Lee *et al.* 2008) and is termed accuracy. Many factors affect the accuracy of WGP, such as marker density, population size, underlying trait genetic architecture, relatedness between training and validation individuals, and prediction model (Zhang *et al.* 2011b; Daetwyler *et al.* 2013; de los Campos *et al.* 2013a). Among all the aspects in WGP, genetic architecture of the complex trait under consideration is known as one influential factor affecting not only the accuracy of WGP but also the relative superiority of different prediction methods (Daetwyler *et al.* 2010; Hayes *et al.* 2010; Riedelsheimer *et al.* 2012b; Daetwyler *et al.* 2013). Both empirical (Eckert *et al.* 2010; Hayes *et al.* 2010; Daetwyler *et al.* 2013) and simulation (Daetwyler *et al.* 2010; Zhang *et al.* 2011a) studies evidenced the robustness of the predictive ability of the genomic best linear unbiased prediction (GBLUP) method (Vanraden 2008) in the prediction of complex traits in highly related populations, which is the typical situation in agricultural populations. When major genes or even genes with moderate effects exist, however, variable selection models, such as BayesB (Meuwissen *et al.* 2001), can predict genomic estimated breeding values (GEBVs) more accurately (Daetwyler *et al.* 2013). Therefore, GBLUP can generally be considered optimal with traits in compliance with the classical infinitesimal model, while models based on variable selection such as BayesB are considered preferable for traits with a genetic architecture including large or moderate effect quantitative trait loci (QTL) (Wimmer *et al.* 2013).

If the genetic architecture of a trait under consideration is unknown, it is hard to choose a proper WGP approach without a serial of laborsome model comparisons. These situations are quite common, such as with traits of low heritability, populations of small size, and novel traits with unknown genetic architecture. This increases the computing burden and uncertainty of implementation for WGP. Hence, a sophisticated model which blends the robustness of GBLUP and the advantage of BayesB appears attractive from an implementation point of view.

Previously, we proposed a genomic BLUP model in which the realized relationship matrix (**G** matrix) was replaced with a trait-specific variance-covariance matrix denoted as **TA** in (Zhang *et al.* 2010), and denoted as **T** hereinafter. Following this, several approaches were proposed to enhance the predictive ability of WGP or the performance of GWAS by building the matrix **T** with different strategies. A weighted GBLUP model was reported to have greater predictive ability compared with GBLUP on complex human traits (de los Campos *et al.* 2013b), in which the **G** matrix was weighted with rescaled *P*-values calculated in a GWAS. Within the framework of single step procedure (Misztal *et al.* 2009; Aguilar *et al.* 2010), an “iterated-GBLUP” algorithm was proposed for genome-wide association study (GWAS) purpose by building a trait-specific **G** matrix weighted by derived marker effects within the iteration procedure (Wang *et al.* 2014). In Zhang *et al.* (2014), the publicly available GWAS / QTL mapping results were used as prior weights to build a **T** matrix in the BLUP-given genetic architecture (BLUP|GA) approach. Similarly, a systems genomic BLUP model, including two **G** matrices built with markers with known or unknown biological function, was proposed theoretically, which can account for and differentiate SNPs with known biological roles in the phenotypic or disease outcomes (Kadarmideen 2014). All previous results support the fact that using a trait-specific variance-covariance matrix can be a useful way to improve the performance of the relevant models, though the optimal way to build **T** is yet to be investigated.

The approach suggested in this study is based on BLUP|GA proposed by Zhang *et al.* (2014) and uses a trait-specific variance-covariance matrix **T** (Zhang *et al.* 2010). Different from Zhang *et al.* (2010), the trait-specific variance-covariance matrix **T** used in this study is a weighted sum of the general realized relationship matrix **G** and a relationship matrix **S** built based on prior information on genetic architecture. While in Zhang *et al.* (2014) the genetic architecture part in **S** was built based on information reported in public GWAS databases, we now present an approach how **S** can be constructed using only information extracted from the dataset at hand. Then, we will investigate the performance of the new strategy with three model traits observed in the German Holstein dairy cattle population. Additionally, to validate the feasibility of the new strategy of BLUP|GA in varying genetic architectures, we will apply the approach on three publicly available common datasets. Finally, the performance of BLUP|GA will be compared with benchmark genomic selection approaches, and features of a trait-specific matrix and the reason why it can improve WGP accuracy will be discussed.

## Materials and Methods

### WGP models

Animal breeders have been using the Henderson’s mixed model equations (MMEs) to predict the breeding values of selection candidates for decades (Henderson 1975a). Conventionally, the variance-covariance structure of additive genetic effects among all individuals was described by the pedigree-based numerator relationship matrix **A** (Henderson 1975b). Recently, with the availability of high-density genetic markers covering the whole genome, a marker-based genetic relationship matrix (generally termed as **G** matrix) is used to replace the **A** matrix in MME (Eding and Meuwissen 2001; Vanraden 2008; Yang *et al.* 2010). The statistical model for best linear unbiased prediction (BLUP) using marker-based genetic relationship matrix can be written as

y=Xμ+Zu+e, (1)

in which **y** is a vector of phenotypic values; μ is the overall mean; **u** is a vector of additive genetic values for all individuals in the model which is assumed to be multivariate normal $u\sim N(0,\sigma_{u}^{2}G)$ with $\sigma_{u}^{2}$ being the additive genetic variance, and **G** the genomic relationship matrix for all individuals (Vanraden 2008); **e** is the residual term with $e\sim N(0,\sigma_{e}^{2}I)$, where $\sigma_{e}^{2}$ is the residual variance; **X** and **Z** are incidence matrices relating the overall mean and additive genetic values to the phenotypic records. The MME corresponding to model (1) is

$$\left[\begin{array}{cc} X^{\text{T}}X & X^{\text{T}}Z\\ Z^{\text{T}}X & Z^{\text{T}}Z+\lambda_{u}G^{-1} \end{array}\right]\cdot \left[\begin{array}{c} \hat{\mu }\\ \overset{⌢}{u} \end{array}\right]=\left[\begin{array}{c} X^{\text{T}}y\\ Z^{\text{T}}y \end{array}\right]$$, (2)

in which $\lambda_{u}=\frac{\sigma_{e}^{2}}{\sigma_{u}^{2}}$. Following previous studies (Vanraden 2008; Hayes *et al.* 2009), the **G** matrix in our study is defined as

$$G=\frac{MM^{T}}{2\sum_{i=1}^{m}p_{i}(1-p_{i})}$$, (3)

where **M** is an adjusted marker genotype matrix including *m* SNPs in columns and *n* individuals in rows. Here, the genotypes are coded as 0, 1, and 2 representing the copy number of the second allele, and then adjusted by 2*p_i_* in each column, where *p_i_* is the allele frequency of the second allele at the *i*th locus in the base population. Since the use of different allele frequencies *p_i_* does not affect the accuracy of prediction (Stranden and Christensen 2011) we use uniformly *p_i_* = 0.5 for all SNPs to build all genomic relationship matrices as in former studies (Zhang *et al.* 2014). The approach using only the **G** matrix defined as in equation (3) was named GBLUP.

In a previous study (Zhang *et al.* 2014), we showed that incorporating GWAS results into WGP via an approach termed BLUP|GA outperforms GBLUP in many situations. Although we use the same BLUP|GA model as in Zhang *et al.* (2014) in the present study, the GA information (**S** matrix) now is extracted from the dataset at hand, rather than from prior GWAS results in the public domain. To run BLUP|GA, in the present study the **G** matrix in the standard MMEs (equation 2) was replaced by a trait-specific **T** matrix, which was calculated as

T=ωS+(1−ω)G, (4)

where ω is an overall weight for the genetic architecture part (**S** matrix), which is a key parameter in BLUP|GA, and denoted as ‘*weight*’ in the following. The **S** matrix used in the present study includes the following information sources: (1) a proportion of SNPs (‘top SNPs’, *top%*) selected according to their size of effect estimated in the training population; (2) possibly the *n* SNPs adjacent (right and left) to each of the top SNPs (*nflank*), which are included to account for linkage disequilibrium between the top SNP and its flanking SNPs; and (3) the corresponding sizes of marker effects for all selected SNPs. Correspondingly, the **S** matrix was defined as

$$S=\frac{M_{1}DM_{1}{}^{\text{T}}}{2\sum_{i=1}^{m_{1}}p_{i}(1-p_{i})}$$, (5)

in which, **M**_1_ represents the genotype matrix of *m*_1_ selected SNPs (top SNPs and their adjacent SNPs), which is a subset of **M**. **D** is a diagonal matrix, and its diagonal elements diag(**D**) contain as weights the estimated marker effects for the selected SNPs in **M**_1_. To ensure analogy of **S** and **G**, diag(**D**) was rescaled such that the mean of diag(**D**) was 1.

By using an equivalent algorithm as proposed by Strandén and Garrick (2009), the marker effect estimates from RRBLUP (ridge regression BLUP) and the genetic values **u** from GBLUP can be obtained in one procedure, which facilitates our top SNP selection, **T** matrix building and GEBV solving. A combined AI-EM restricted maximum likelihood algorithm (AI-average information, EM-expectation maximization) was used to estimate variance components ($\sigma_{{}_{\text{T}}}^{2}$, $\sigma_{\text{u}}^{2}$, and $\sigma_{e}^{2}$) via the DMU software package (Madsen *et al.* 2006). To run BLUP|GA, three parameters *weight* (*ω* in equation 4), *top%* (a proportion of top SNPs), and *nflank* (*n* SNPs adjacent to each of the top SNPs) need to be assigned. Both *weight* and *top%* range from 0 to 1, and 0 was assigned to *nflank* as default value accompanied with a maximum value of 10 in our calculation. In this study, we tentatively used a grid search strategy to identify the optimal parameters.

The full population usually includes a reference population and a candidate population. The entire procedure of using BLUP|GA includes two stages: a training stage by obtaining the optimal parameters from the reference population, and an application stage by applying the identified optimal parameters to the candidate population. Within the training stage, the reference population is divided as training and validation set to conduct cross validation (CV), hence ‘folds’ and ‘replicates’ were used in this stage. The algorithm used in this study is as follows:

1. Build **G** matrix with equation (3), calculate $\hat{s}=Z{\left[G+\lambda_{u}I\right]}^{-1}Z^{T}(y-X\hat{b})$, where $\hat{b}$ is the generalized least-square solution for fixed effect **b** (the overall mean μ in eq.(2)), $\hat{b}={\left(X^{\text{T}}Z{\left[G+\lambda_{u}I\right]}^{-\text{1}}Z^{\text{T}}X\right)}^{-\text{1}}X^{\text{T}}Z{\left[G+\lambda_{u}I\right]}^{-\text{1}}Z^{\text{T}}y$, and $\lambda_{u}=\frac{\sigma_{\text{e}}^{2}}{\sigma_{u}^{2}}$ as defined in equation (2);
2. Calculate the additive genetic values of GBLUP $\hat{u}=GZ^{\text{T}}Z{\left[G+\lambda_{u}I\right]}^{-1}Z^{\text{T}}(y-X\hat{b})=GZ^{\text{T}}\hat{s}$ (optional step, run this step only when standard GBLUP solutions are needed);
3. Calculate the marker effects (RRBLUP solution)
  $$\hat{g}=\sigma_{\text{g}}^{2}\sigma_{\text{u}}^{-2}M^{\text{T}}Z^{\text{T}}Z^{}{\left[G+\lambda_{u}I\right]}^{-1}Z^{T}(y-X\hat{b})=\sigma_{\text{g}}^{2}\sigma_{\text{u}}^{-2}M^{\text{T}}Z^{\text{T}}\hat{s}$$,
  where $\sigma_{\text{g}}^{2}=\sigma_{\text{u}}^{2}/\sum_{i=1}^{m}2p_{i}(1-p_{i})$, as defined in Strandén and Garrick (2009);
4. Given *top%*, *nflank*, and ω, select the SNPs with the largest size of marker effect $\hat{g}$ (top SNPs), build **M**_1_ and **D**, build $S=\frac{M_{1}DM_{1}{}^{\text{T}}}{2\sum_{i=1}^{m_{i}}p_{i}(1-p_{i})}$, build T=ωS+(1−ω)G;
5. Calculate ${\hat{s}}^{*}=Z{\left[T+\lambda_{\text{T}}I\right]}^{-1}Z^{\text{T}}(y-X{\hat{b}}^{*})$, and get BLUP|GA solution ${\hat{u}}^{*}=TZ^{\text{T}}Z^{}{\left[T+\lambda_{\text{T}}I\right]}^{-1}Z^{\text{T}}(y-X{\hat{b}}^{*})=TZ^{\text{T}}{\hat{s}}^{*}$, where $\lambda_{\text{T}}=\frac{\sigma_{\text{e}}^{2}}{\sigma_{\text{T}}^{2}}$;
6. Given different *top%*, *nflank*, and ω, repeat steps 4 and 5 to predict the GEBVs of validation set, which is also a grid search of the optimal parameters for BLUP|GA within the current fold;
7. Repeat steps 1−6 within a cross-validation procedure by providing different folds of datasets and obtain the global optimal parameters by maximizing the average accuracy across all folds/replicates within the reference population;
8. Apply the globally optimal parameters obtained from reference population to the full population to predict GEBVs of candidate population (never used in steps 1−7) by running steps 1−5 once.

The aforementioned algorithm obtains the optimal parameters from the reference population by applying CV and grid search via steps 1–7 (denoted as “training stage” in the sections to follow), and applies these parameters to the candidate population via step 8 (denoted as “application stage” in the following). It should be noted that the steps 1–5 are the core algorithm of BLUP|GA. Given a dataset, and given all parameters, GEBV solutions for BLUP|GA can be obtained from these five steps. It will not matter whether this dataset is a training / reference or both training / reference and validation / candidate set, since the matrix **Z** can indicate it correctly.

### The German Holstein genomic prediction population

A German Holstein genomic prediction population comprising 5024 bulls, provided by Vereinigte Informationssysteme Tierhaltung w.V., was used to validate our new strategy based on the BLUP|GA approach. All bulls were genotyped with the Illumina Bovine SNP50 Beadchip (Matukumalli *et al.* 2009). After quality control, 42,551 SNPs remained for our further analyses. Highly reliable conventional estimated breeding values (EBVs) of three traits, milk fat percentage (FP), milk yield (MY), and somatic cell score (SCS), were available for all bulls. Statistics of the phenotypes are shown in Table 1. These three traits were used in the present study because of the well-established knowledge from previous studies (Hu *et al.* 2013; Zhang *et al.* 2014) and their representative distinct genetic architectures. They may represent three genetic architectures of complex traits that are composed of (1) one major gene and a large number of small effect loci (FP), (2) few moderate effect loci and many small effect loci (MY), and (3) many loci with small effects (SCS), respectively. EBVs of the three traits were used as “phenotypes” in our WGP model. To consider the performance of WGP methods in smaller population size, we randomly selected subsets of 4000, 2000, 1000, 500, 250, and 125 bulls from the full dataset, respectively. This datasets are available in File S1 and File S2.

**Table 1:** Summary of datasets

Dataset	Trait	N	Mean*^a^*	SD*^a^*	r^2^/h^2^*^b^*
Cattle	Fat percentage	5024	−0.06	0.28	0.94
	Milk yield	5024	370.79	641.60	0.95
	Somatic cell score	5024	102.32	11.73	0.88
Loblolly pine	Rustbin	807	−0.01	0.40	0.21
	Gall	807	−0.02	1.13	0.12
	Density	910	0.05	2.50	0.09
	Rootnum	925	0.32	0.96	0.07
	CWAC	861	2.28	42.03	0.45
	Rootnum_bin	925	0.11	0.26	0.10
QTL-MAS2012	T1	3000	0.00	176.52	0.36
	T2	3000	0.00	9.51	0.35
	T3	3000	0.00	0.02	0.52
GSA data	PolyUnres	2000	−	−	0.25
	GammaUnres	2000	−	−	0.25
	PolyRes	2000	−	−	0.25
	GammaRes	2000	−	−	0.25

a Mean and (SD) of conventional estimated breeding values for the three cattle traits or phenotypic values for other traits; we did not calculate the statistics for GSA data because it includes 10 replicates of the simulated datasets. GSA, Genetics Society of America

b Reliability (*r*^2^) for cattle trait estimated breeding value, or heritability (*h*^2^) for other trait phenotypes.

### Public datasets

Besides the dairy cattle dataset, we analyzed three additional publicly available datasets for model validation, (1) the loblolly pine dataset (Resende *et al.* 2012), (2) the livestock dataset simulated by Hickey and Gorjanc (2012), and (3) the common dataset provided by the 2012 QTL-MAS Workshop (Usai *et al.* 2012). The first two datasets were chosen because they were recommended by the Genetics Society of America (GSA) for genomic selection model comparison (de Koning and McIntyre 2012). Because these two datasets were analyzed by many researchers with a variety of WGP models before, they serve as a good reference for method comparisons. The third dataset was chosen because it was also analyzed in detail by all participants of the 16th QTL-MAS Workshop and results were compared by organizers (Usai *et al.* 2012).

#### Loblolly pine dataset:

The loblolly pine genomic selection dataset provided by Resende *et al.* (2012) comprises 951 individuals from 61 families with each individual systemically phenotyped for 17 traits. These individuals were genotyped with an Illumina Infinium assay (Illumina, San Diego, CA) originally designed with 7216 SNPs in a previous study (Eckert *et al.* 2010). A subset of 4853 polymorphic SNPs was used in Resende *et al.* (2012) as well as in the present study. Among the 17 traits, we selected 6 traits according to the results in Resende *et al.* (2012): four traits (Rust_bin, Rust_gall_vol, Density, and Rootnum) for which advantages of using BayesA or BayesCpi were found and two additional traits (CWAC and Rootnum_bin) were selected due to their representative heritabilities (highest and lowest in the remaining 13 traits).

#### GSA-simulated dataset:

This simulated livestock dataset (Hickey and Gorjanc 2012) comprised 2000 individuals in generations 4 and 5 (1000 individuals per generation) as reference population, and 1500 individuals in generation 6, 8, and 10 (500 individuals per generation) as candidate population. In this simulation, ~1.67 million segregating sites were generated, and within these sites 60,000 and 9000 sites were randomly selected as SNP markers and candidate QTL, respectively. The 9000 candidate QTL were selected with minor allele frequencies <0.3 or without restriction to their minor allele frequencies. The additive genetic effects of all candidate QTL was sampled from either a standard normal distribution or a gamma distribution [shape parameter = 0.4, scale parameter = 1.66 (Meuwissen *et al.* 2001)]. Hence, phenotypes on four traits, denoted by PolyUnres, GammaUnres, PolyRes, and GammaRes, for each individual in the reference population were generated with a heritability of 0.25. We predicted the genetic merit of each candidate individual using phenotypic data from reference individuals within each of the 10 replicates of the simulated datasets (Hickey and Gorjanc 2012).

#### QTL-MAS 2012 common dataset:

The third public dataset we analyzed is the 16th QTL-MAS Workshop dataset simulated by Usai *et al.* (2012), which can be downloaded from http://qtl-mas-2012.kassiopeagroup.com/en/dataset.php. It includes 3000 training individuals in generation 1−3 and 1000 validation individuals in generation 4. The genome comprises five chromosomes and 4000 SNPs per chromosome. To generate the genetic effect, 50 QTL were selected randomly with their effects drawn from a gamma distribution (shape parameter = 0.42, scale parameter = 5.4). Three genetically correlated quantitative trait phenotypes were generated based on the 50 QTL for each of the 3000 training individuals. We predicted the genetic merit for 1000 validation individuals using the data from the training individuals.

### Model validation and accuracy

For the dairy cattle dataset, the full dataset was divided into two subsets, a reference population with randomly selected N bulls (N = 125, 250, 500, 100, 2000, and 4000), and a candidate population with the rest (5024-N) bulls. In the training stage, a fivefold cross-validation procedure (Stone 1974) was conducted to compare the predictive ability of different WGP models and to obtain the optimal parameters for BLUP|GA in the reference population. In a fivefold CV, we randomly divided the reference individuals into five groups (folds) with equal size and used four folds to train the model and to predict the GEBVs of the fifth fold. This was repeated five times, so that individuals in each of the five folds were predicted once. The fivefold CV was repeated 20 times, resulting in 20 averaged results for predictive abilities or unbiasedness, respectively. In the application stage, optimal parameters obtained from training stage were applied to the full dataset (both reference and candidate) to assess the predictive ability of BLUP|GA and other WGP methods in the candidate population. This was done to mimic the practical situation. The accuracy for this dataset was defined as the correlation of GEBV with conventional EBV in the validation set in the training stage or conventional EBV in the candidate population in the application stage, r=cor(GEBV,EBV). The regression of GEBV on EBV, b=reg(GEBV,EBV), was used to assess the unbiasedness.

For the three public datasets, different validation procedures were used to make the results from the present study being comparable with those reported in the original studies, in which these public datasets were analyzed with several WGP methods (Resende *et al.* 2012; Usai *et al.* 2012; Daetwyler *et al.* 2013).

#### Loblolly pine dataset:

For the loblolly pine dataset, one replicate of 10-fold CV was conducted to validate the BLUP|GA method, and hence only results from the training stage were reported. Similar validation procedures were used to assess several methods, such as BayesA, BayesCpi, RRBLUP, and BLASSO, in Resende *et al.* (2012). The accuracy for this dataset was defined as the correlation of GEBV with deregressed phenotypes (*DP*) r=cor(GEBV,DP).

#### GSA common dataset:

In each of the 10 GSA common datasets (Hickey and Gorjanc 2012), the genetic merit of the candidate individuals was predicted from the reference set using nine GS methods as presented in the review article of (Daetwyler *et al.* 2013). We assessed the BLUP|GA with this dataset in the same manner as (Daetwyler *et al.* 2013). The accuracy was determined as the correlation between GEBV and simulated true breeding values (*TBV*) r=cor(GEBV,TBV) in the candidate sets.

#### QTL-MAS 2012 dataset:

The 2012 QTL-MAS Workshop common dataset includes only one reference population and one candidate population (Usai *et al.* 2012). Hence, it is suitable to demonstrate the entire calculation strategy of BLUP|GA with this dataset. A fivefold cross validation procedure with 10 replicates was conducted within the reference population to obtain the optimal parameters for BLUP|GA, and then these parameters were applied to predict GEBV for the candidate population. The performance both in the training stage and the application stage are reported. Accuracy in this dataset was defined as the correlation between GEBV and simulated TBV in the validation set of the training stage or in the candidate set of the application stage, r=cor(GEBV,TBV).

## Results

### GEBV accuracies and marker effects in the dairy cattle dataset

**Figure 1:**
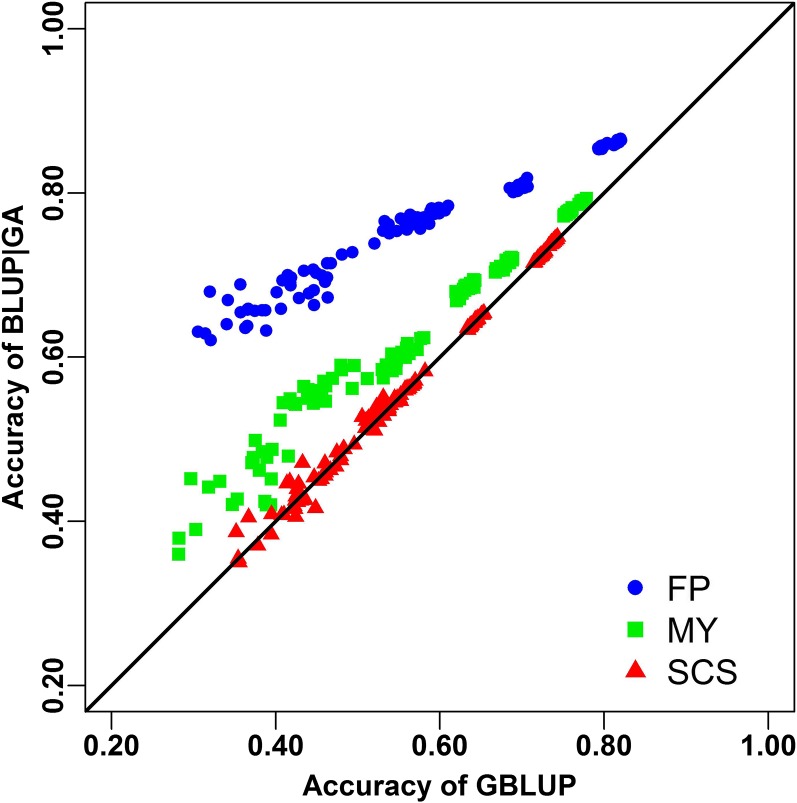
Accuracy of genomic prediction using genomic best linear unbiased prediction (GBLUP) and BLUP-given genetic architecture (BLUP|GA). Points showed the average accuracies of each fivefold cross validation from scenarios using different population sizes. Results for fat percentage (FP), milk yield (MY), and somatic cell score (SCS) are presented with blue filled cycles, green filled squares, and red filled triangles, respectively.

We first validated BLUP|GA in the dairy cattle population with 5024 bulls (Table 1) via two validation procedures. For the three validated traits (FP, MY, and SCS), the accuracy and unbiasedness of BLUP|GA and GBLUP, obtained from the mean of 20 replicates of fivefold cross-validation (training stage), are shown in Figure 1 and Table 2. The accuracy of BLUP|GA and GBLUP in the candidate population calculated from the application stage and parameters for *weight*, *top%* and *nflank* used for the application stage and derived from the training stage are shown in Table 3. For scenario N = 5024, *i.e.*, all available individuals were used in the training stage, only the optimal parameters derived from the reference population are reported. In both validation procedures, the trend and size of the advantage of BLUP|GA over GBLUP is consistent. BLUP|GA dominated GBLUP for FP and MY, and the advantage decreased with an increased size of the reference population (Table 2 and Table 3), which suggests that using “correct” prior information is particularly important for small datasets, as also noted by de los Campos *et al.* (2013a). By using BLUP|GA with the optimal set of parameters in a small population (N = 125), the accuracies of genomic prediction were increased by 82.2% and 23.8% in the training stage, and 113.4% and 30.8% in the application stage, for FP and MY, respectively (Table 2 and Table 3). An advantage of 5.6% and 1.9% in the training stage could still be observed for the two traits even if we used the whole population (N = 5024, Table 2). BLUP|GA did not consistently outperform GBLUP for SCS in any of the scenarios investigated (Figure 1, Table 2), which suggests that a zero *weight* should be assigned to **S** for this trait in BLUP|GA. With respect to the unbiasedness, both BLUP|GA and GBLUP performed well for all scenarios with this dataset. The optimal parameters applied to BLUP|GA are provided in Table 3. Generally, the optimal **S** matrix in this dataset was built with a small proportion of top SNPs and five adjacent SNPs on right and left, respectively. While for all traits between 0.01 and 0.5% of the top SNPs were accounted for—with a tendency toward a smaller proportion in smaller reference sets—the average optimal *weight* assigned to those selected SNPs was 32, 4.0, and 2.0% for FP, MY, and SCS, respectively (Table 3).

**Table 2:** Accuracy and unbiasedness of genomic prediction in the dairy cattle dataset from training stage

*N*	Method	Fat Percentage		Milk Yield		Somatic Cell Score	
		*r*_(EBV, GEBV)_*^a^*	*b*_(EBV,GEBV)_ *^b^*	*r*_(EBV, GEBV)_	*b*_(EBV,GEBV)_	*r*_(EBV, GEBV)_	*b*_(EBV,GEBV)_
5024	GBLUP	0.816 ± 0.000*^c^*	1.003 ± 0.001	0.774 ± 0.001	1.010 ± 0.001	0.738 ± 0.001	0.996 ± 0.001
	BLUP|GA	0.862 ± 0.000	0.959 ± 0.001	0.789 ± 0.000	0.990 ± 0.001	0.741 ± 0.001	0.981 ± 0.001
4000	GBLUP	0.798 ± 0.001	1.007 ± 0.001	0.757 ± 0.001	1.011 ± 0.001	0.722 ± 0.001	1.001 ± 0.001
	BLUP|GA	0.856 ± 0.000	0.965 ± 0.001	0.777 ± 0.001	0.990 ± 0.001	0.723 ± 0.001	1.001 ± 0.001
2000	GBLUP	0.698 ± 0.001	0.997 ± 0.002	0.680 ± 0.001	1.014 ± 0.002	0.642 ± 0.001	1.005 ± 0.002
	BLUP|GA	0.808 ± 0.001	0.963 ± 0.002	0.714 ± 0.001	0.992 ± 0.002	0.643 ± 0.001	0.996 ± 0.002
1000	GBLUP	0.594 ± 0.002	1.005 ± 0.004	0.632 ± 0.002	1.072 ± 0.003	0.555 ± 0.003	1.019 ± 0.006
	BLUP|GA	0.778 ± 0.001	0.978 ± 0.002	0.683 ± 0.002	1.039 ± 0.002	0.556 ± 0.003	1.008 ± 0.006
500	GBLUP	0.557 ± 0.004	1.102 ± 0.008	0.551 ± 0.004	1.151 ± 0.009	0.526 ± 0.004	1.128 ± 0.009
	BLUP|GA	0.761 ± 0.002	0.983 ± 0.003	0.600 ± 0.003	1.051 ± 0.007	0.531 ± 0.004	1.098 ± 0.008
250	GBLUP	0.441 ± 0.006	1.111 ± 0.016	0.447 ± 0.007	1.230 ± 0.018	0.441 ± 0.008	1.157 ± 0.024
	BLUP|GA	0.697 ± 0.004	0.952 ± 0.006	0.555 ± 0.006	1.087 ± 0.011	0.435 ± 0.007	1.058 ± 0.022
125	GBLUP	0.371 ± 0.010	1.108 ± 0.032	0.361 ± 0.010	1.167 ± 0.040	0.424 ± 0.009	1.328 ± 0.030
	BLUP|GA	0.676 ± 0.005	0.959 ± 0.011	0.447 ± 0.010	1.168 ± 0.030	0.435 ± 0.009	1.257 ± 0.026

EBV, estimated breeding value; GEBV, genomic estimated breeding value; GBLUP, genomic best linear unbiased prediction; BLUP|GA, best linear unbiased prediction-given genetic architecture.

a Accuracies (*r*) were calculated as the correlation between the conventional EBV and the GEBV in the validation set in cross validation procedure.

b Unbiasednesses (*b*) were calculated as the regression coefficient of the conventional EBV on the GEBV in the validation set.

c The mean (± SE) of the 20 averaged accuracies from each replicates of fivefold cross-validation.

**Table 3:** Accuracy of BLUP|GA and GBLUP and the optimal parameters used in the application stage in the dairy cattle dataset

Trait	N*^a^*	Accuracy*^b^*		BLUP|GA Parameters		
		GBLUP	BLUP|GA	*top%**^c^*	*weight**^d^*	*nflank**^e^*
Fat percentage	125	0.321	**0.685**^*f*^	0.01	0.44	3
	250	0.417	**0.714**	0.01	0.68	3
	500	0.508	**0.734**	0.01	0.40	3
	1000	0.629	**0.768**	0.05	0.20	3
	2000	0.734	**0.813**	0.50	0.16	5
	4000	0.796	**0.845**	0.50	0.18	5
	5024	–	–	0.50	0.18	5
Milk yield	125	0.370	**0.484**	0.01	0.02	5
	250	0.449	**0.523**	0.01	0.04	5
	500	0.549	**0.609**	0.01	0.02	5
	1000	0.638	**0.677**	0.05	0.04	5
	2000	0.717	**0.740**	0.05	0.02	5
	4000	0.767	**0.774**	0.50	0.04	5
	5024	–	–	0.10	0.10	5
Somatic cell score	125	**0.317**	0.295	0.01	0.02	5
	250	**0.433**	0.419	0.01	0.02	5
	500	**0.528**	0.520	0.05	0.02	5
	1000	0.596	**0.596**	0.10	0.02	5
	2000	0.665	**0.669**	0.50	0.02	5
	4000	0.731	**0.736**	0.50	0.02	5
	5024	–	–	0.50	0.04	5

BLUP|GA, best linear unbiased prediction-given genetic architecture; GBLUP, genomic best linear unbiased prediction; EBV, estimated breeding value; GEBV, genomic estimated breeding value; SNP, single-nucleotide polymorphisms.

a Size of the reference population.

b Accuracy is calculated as the correlation between the conventional EBV and GEBV of GEBV in the candidate population with population size of 5024 - N.

c Percentage of top SNPs.

d Overall weight ω for the genetic architecture part while building **T** matrix.

e Number of selected flanking SNPs near each top SNPs.

f Scenario with higher accuracy is shown in bold face.

**Figure 2:**
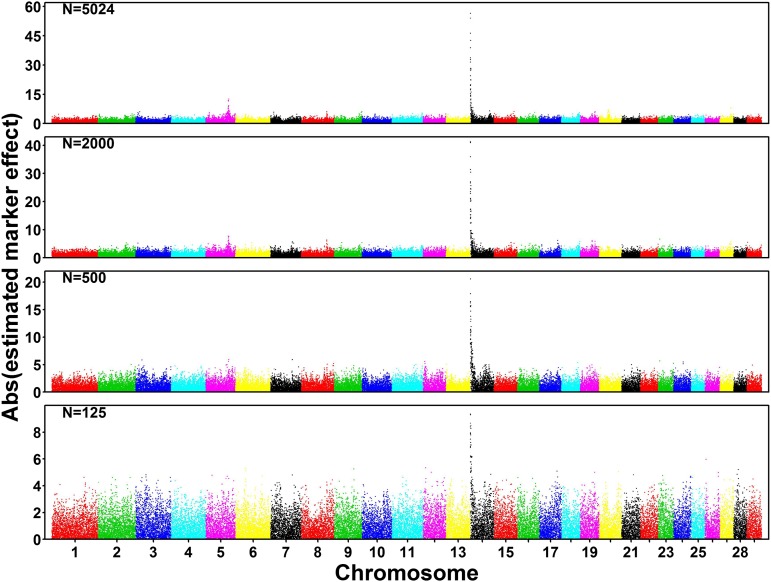
Manhattan plot of the marker effects estimated for fat percentage. The marker effects (*g*_i_) were estimated using ridge regression best linear unbiased prediction and rescaled so that the average marker effect was 1, in order to make the sizes of marker effect from different population sizes (N) or different traits comparable.

In addition, we compared the marker effects estimated from different population sizes for fat percentage (Figure 2), milk yield (Supporting Information, Figure S1), and somatic cell score (Figure S2), respectively. To ensure that the effects estimated from different traits and / or different population sizes were comparable, we rescaled the marker effects so that the average absolute value of marker effect was 1 for each scenario. It is clear that the marker with the highest estimated effect was found around DGAT1 (Diacylglycerol O-Acyltransferase 1, the peak in the left side of chromosome 14) for all scenarios in fat percentage (Figure 2) and milk yield (Figure S1). Although decreasing the population size (*N*) from 5024 to 125, the scaled peak value decreased from 60 to 9 for fat percentage (Figure 2), from 26 to 7 for milk yield (Figure S1), but no apparent decrease was observed for SCS (Figure S2). Similar effects on both the size and variance of SNP effects were reported in Liu *et al.* (2011) for a German Holstein dataset.

### GEBV accuracies in public datasets

To validate BLUP|GA in a variety of applications, we additionally analyzed three common datasets: loblolly pine dataset (Resende *et al.* 2012), GSA-simulated dataset (Hickey and Gorjanc 2012), and 2012 QTL-MAS Workshop dataset (Usai *et al.* 2012) (Table 1). The accuracy for GBLUP and BLUP|GA calculated in this study and the optimal parameters for BLUP|GA are listed in Table 4. The parameters include the proportion of selected top SNPs (*top%*), overall weights assigned to the **S** matrix (*weight*), and the number of flanking markers selected accompanied with each top SNP (*nflan*k). The reported accuracies achieved by the genomic selection methods BayesA, BayesB, BayesC, and RRBLUP in other studies are shown in Table 5.

**Table 4:** Accuracy and optimal parameters of BLUP|GA for common datasets obtained from the training stage

Dataset	Trait	Accuracy		BLUP|GA Parameters		
		GBLUP	BLUP|GA	*top%**^a^*	*weight**^b^*	*nflank**^c^*
Loblolly pine	Rustbin	0.298	**0.385**^*d*^	0.12	0.140	0
	Gall	0.237	**0.346**	0.32	0.450	0
	Density	0.238	**0.241**	5.00	0.024	0
	Rootnum	0.268	**0.270**	5.20	0.024	0
	CWAC	0.475	**0.478**	0.15	0.006	0
	Rootnum_bin	**0.288**	**0.288**	0.25	0.005	0
QTL-MAS2012	T1	0.707	**0.779**	0.40	0.280	5
	T2	0.717	**0.802**	0.20	0.300	5
	T3	0.761	**0.847**	0.20	0.600	5
GSA dataset	PolyUnres	0.453	**0.454**	5.00	0.010	2
	GammaUnres	0.442	**0.546**	0.12	0.123	2
	PolyRes	0.390	**0.391**	6.00	0.010	2
	GammaRes	0.410	**0.491**	0.17	0.175	2

BLUP|GA, best linear unbiased prediction-given genetic architecture; GBLUP, genomic best linear unbiased prediction; GSA, Genetics Society of America; SNP, single-nucleotide polymorphism.

a Percentage of top SNPs.

b Overall weight ω for the genetic architecture part while building **T** matrix.

c Number of selected flanking SNPs near each top SNPs, the *nflank* was set to 0 for Loblolly and not chosen in a validation procedure.

d Scenario with the highest accuracy is shown in bold face.

**Table 5:** Accuracy for different genomic selection models in the three validation datasets

Dataset	Trait	GBLUP	BLUP|GA*^a^*	BayesA*^b^*	BayesB*^b^*	BayesC*^b^*	RRBLUP*^b^*
Loblolly pine	Rustbin	0.298	**0.385**^*c*^	0.34	–	0.34	0.29
	Gall	0.237	**0.346**	0.28	–	0.29	0.23
	Density	0.238	**0.241**	0.23	–	0.22	0.20
	Rootnum	0.268	**0.270**	0.25	–	0.24	0.24
	CWAC	0.475	**0.478**	0.47	–	0.47	**0.48**
	Rootnum_bin	**0.288**	**0.288**	0.27		0.28	0.28
QTL-MAS2012	T1	0.732	**0.797**	0.794	0.794	–	0.707
	T2	0.771	**0.843**	0.834	0.834	–	0.746
	T3	0.758	**0.838**	0.828	0.828	–	0.723
GSA dataset	PolyUnres	0.453	**0.454**	0.453	0.451	0.452	0.453
	GamUnres	0.442	**0.546**	0.539	0.544	0.542	0.447
	PolyRes	0.390	**0.391**	0.388	0.383	0.390	0.390
	GammaRes	0.410	0.491	0.495	0.504	**0.505**	0.413

GBLUP, genomic best linear unbiased prediction; BLUP|GA, best linear unbiased prediction-given genetic architecture.

a Accuracy of BLUP|GA were calculated in the application stage for QTL-MAS2012 dataset and in the training stage for pine and GSA dataset.

b BayesA, BayesB, BayesC, and RRBLUP results were obtained from Table S1 in Daetwyler *et al.* (2013).

c Scenario with the highest accuracy is shown in bold face.

#### Loblolly pine:

For the six selected traits in the loblolly pine dataset, BLUP|GA showed a consistent advantage over other methods (Table 5). We expected that BLUP|GA outperform GBLUP and its equivalent method RRBLUP for the first four traits, because advantages of variable selection methods over GBLUP/RRBLUP have already been observed by Resende *et al.* (2012). Results confirmed our assumption, and BLUP|GA outperformed GBLUP/RRBLUP and even variable selection methods such as BayesA. It should be noted that, for Rustbin and Gall, BLUP|GA increased the accuracy by 29.2% and 46.0% compared with GBLUP, and by 12.6% and 23.6% compared with BayesA (Table 5), respectively. For the remaining two traits (CWAC and Rootnum_bin), BLUP|GA yielded a similar accuracy as GBLUP/RRBLUP (Table 5). It is obvious that the optimal overall weight (*ω*) decreased from trait Gall (Table 4), Rustbin to Rootnum_bin, accompanied with the decreased advantage of BLUP|GA.

#### GSA-simulated dataset:

The GSA-simulated common dataset has been well investigated by Daetwyler *et al.* (2013). The BLUP|GA accuracy is greater than GBLUP accuracy by 0.104 (23.3%) and 0.081 (19.8%) for the trait GammaUnres and GammaRes, respectively (Table 4). BLUP|GA and GBLUP performed equally well on the two polygenic traits, PolyUnres and PolyRes. This result met our expectation since the traits GammaUnres and GammaRes were controlled by a small number of genes (N = 900) (Hickey and Gorjanc 2012), suggesting a characteristic underlying trait genetic architecture, which is not the case for PolyUnres and PolyRes.

Daetwyler *et al.* (2013) provided the accuracy of 10 GS approaches in their Table S1. To make the methods comparison easier, we extracted the comparable results from Daetwyler *et al.* (2013) and showed them in Table 5. When averaged across the four traits, BLUP|GA performed as well as BayesB, slightly underperformed BayesC by 0.002, and outperformed BayesA and RRBLUP by 0.002 and 0.047, respectively.

#### QTL-MAS 2012 dataset:

For the three traits of the 2012 QTL-MAS Workshop dataset, we derived the BLUP|GA model parameters with the 3000 individuals in the reference population by CV (training stage), and then predicted the genetic merit for 1,000 candidate individuals (application stage). In the two stages, accuracy of both GBLUP and BLUP|GA were calculated, respectively (Table 4 and Table 5). According to optimal parameters obtained from training stage (Table 4), only ~0.3% top SNPs and their flanking SNPs were used to build the **S** matrix and finally forming the **T** matrix for each trait. The accuracy of BLUP|GA was 0.779, 0.843, and 0.838 for trait 1, 2, and 3, respectively (Table 5). In addition, we collected the accuracy reported by the 2012 QTL-MAS Workshop organizers from Gaspa *et al.* (2012) and presented them in Table 5 for method comparison. The achieved accuracy from BLUP|GA is the highest among all the approaches including BayesA, BayesB, GBLUP, and RRBLUP. An exception is the accuracy of the approach ‘GLASSO_20’ (proposed by Gaspa *et al.* (2012)) which was 0.853 for trait 2. Although this is slightly greater than the accuracy obtained by BLUP|GA, the accuracy of ‘GLASSO_20’ was 0.778 for trait 1, and was absent for trait 3. Hence we did not compare BLUP|GA with ‘GLASSO_20’ in Table 5. On average across the three traits, BLUP|GA outperformed GBLUP and BayesB by 0.072 (9.6%) and 0.007 (0.9%) in accuracy, respectively (Table 5).

**Figure 3:**
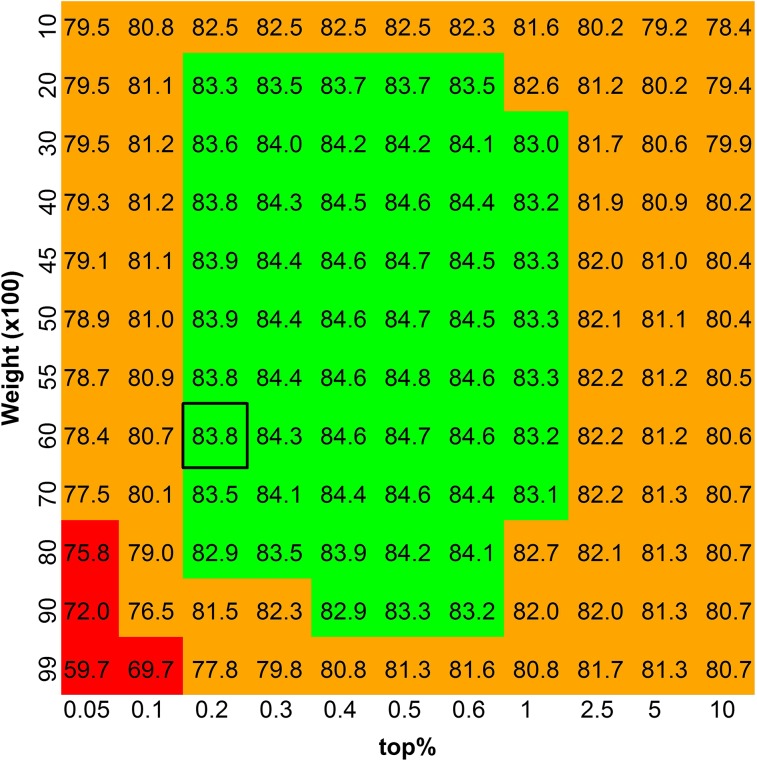
Heat map of the best linear unbiased prediction−given genetic architecture (BLUP|GA) accuracy for trait T3 in the validation population from QTLMAS dataset. The accuracy of best linear unbiased prediction-given genetic architecture (BLUP|GA) (×100) calculated with the assigned weight (vertical axes) and *top%* (horizontal axes) is shown in each cell of the heat map. Red area shows scenarios that BLUP|GA performs worse than genomic best linear unbiased prediction (0.758), green area shows scenarios that BLUP|GA performs better than BayesB (0.828). The optimal parameter combination obtained from reference population by cross validation is shown in black box.

To illustrate the pattern of predictive ability of BLUP|GA with respect to the parameters assigned, the accuracy of BLUP|GA for trait T3 in the candidate population with weights ranging from 0.1 to 0.99, *top%* ranging from 0.05 to 10% and *nflank* = 6 is shown in Figure 3. Given the aforementioned parameters, the accuracy of BLUP|GA ranged from 0.597 (*weight* 0.99, *top%* 0.05%) to 0.848 (*weight* 0.55, *top%* 0.5%). In this calculation, inferiority of BLUP|GA performance was only observed when too large weight was given to a very small proportion of top SNPs, while for a wide range of parameter combinations (*weight* 0.2−0.9%, *top%* 0.2–1.0%) superior performance of BLUP|GA compared to BayesB was observed (Figure 3).

### Genetic variance explained by top SNPs

**Figure 4:**
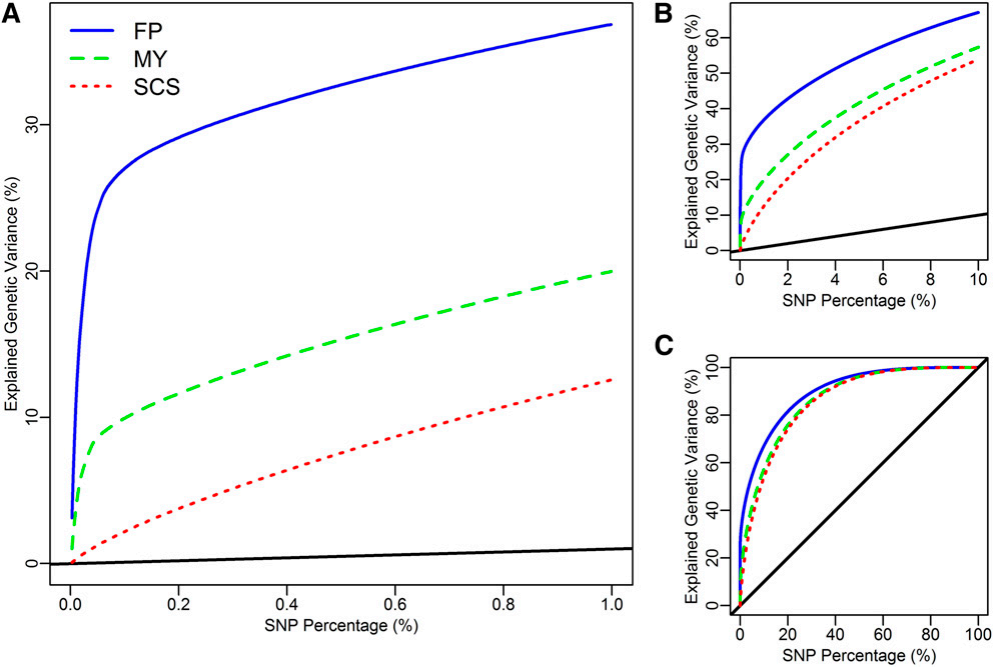
Cumulative proportion of genetic variance explained by single-nucleotide polymorphisms (SNPs). The top 1% (A), 10% (B) and 100% (C) SNPs were sorted by the size of estimated effects in decreasing order. Results for fat percentage, milk yield, and somatic cell score were plotted with blue solid lines, green dash lines and red dotted lines, respectively. The marker weights for genomic best linear unbiased prediction are shown by black solid lines.

Results in Table 2 clearly show that BLUP|GA improved the accuracy for FP and MY, but not for SCS. To determine the feature of a trait on which the accuracy of WGP can be improved, we calculated the genetic variance explained by each marker as $2p(1-p)\alpha^{2}$, where *p* and *α* are the allele frequency and the estimated allele substitution effect for the marker under consideration. Then, we sorted all markers by their size of estimated effects (|*α|*) in decreasing order, and finally plotted the cumulative proportion of genetic variance explained by the ordered SNPs for each scenario. The proportion of genetic variance explained by the top 1%, 10%, and 100% SNPs are shown in panels A, B, and C in Figure 4. Interestingly, the differences among the three curves occur mainly at the top SNPs, especially for the top ~0.1% SNPs (Figure 4A), and the curves are nearly parallel for the remaining part (Figure 4C). For fat percentage, more than one third of the genetic variance is explained by the top 1% SNPs; moreover, only the top 0.1% SNPs (43 SNPs) explain ~28% of the genetic variance. By contrast, the top 1% SNPs explain only ~13% of the genetic variance for somatic cell score (Figure 4A).

Additionally, we analyzed the proportion of genetic variance explained by top SNPs in scenarios with different population sizes (*N*) for all the three model traits in the dairy cattle dataset. By decreasing the population size from 5024 to 125, not only the size of estimated marker effects (Figure 2), but also the proportion of genetic variance explained by top SNPs (Figure S3, Figure S4, and Figure S5) decreased for all three traits. The same trend was observed for milk yield in another study within the German Holstein population (Liu *et al.* 2011). However, the impact of population size differed among traits. For example, while decreasing the population size from 5024 to 125, the genetic variance explained by top 1% SNPs decreased from 36 to 12% for FP (Figure S3), but it changed from 13 to 11% only for SCS (Figure S5).

To validate the explanatory power of the pattern observed in the cattle dataset, we plotted the proportion of genetic variance explained by top SNPs for Rust_bin and Rootnum_bin in the loblolly pine dataset (Figure S6 and Figure S7). By decreasing the population size from 807 to 202 (~25%), the genetic variance explained by top 1% SNPs decreased from 18.5 to 16.4% for rust_bin (Figure S6), but no decrease was observed for rootnum_bin (Figure S7).

### Trait-specific genetic variance-covariance matrix

**Figure 5:**
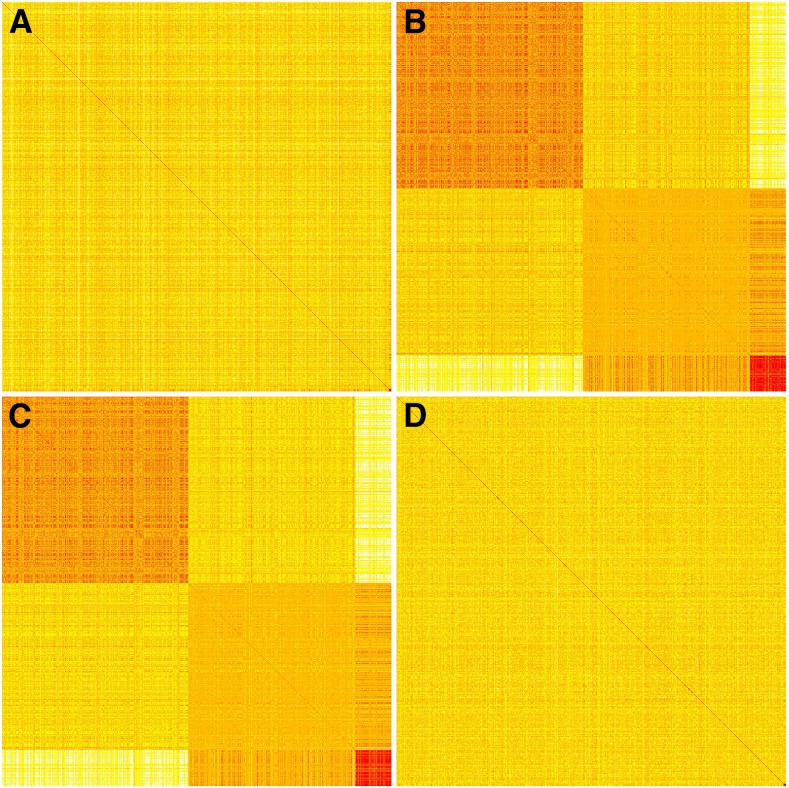
Heat maps of the realized relationship matrix (G) and three trait-specific relationship matrices (S) in dairy cattle dataset. The **G** matrix was built with all markers (A), and **S** matrices were built with top 1% SNPs for fat% (B), milk yield (C), and somatic cell score (D), respectively. These matrices were calculated with the genotypes of 1000 randomly selected bulls, and these bulls were sorted by their genotypes of the SNP with the largest marker effects for each trait.

To investigate the causes of the differential predictive ability of GBLUP and BLUP|GA, we plotted heat maps of the **G** matrix and the three **S** matrices (component of **T** matrix; see the section *Materials and Methods* for details) built for the three traits in the dairy cattle dataset (Figure 5). Individuals in all **S** matrices were ordered by the genotypes of SNP with the largest estimated effect on fat%. The **S** matrix for FP and MY showed apparent blocks (Figure 5, B and C), which is reflecting the three DGAT1 genotypes, and was distinct from the **G** matrix (Figure 5A), whereas the difference between **S** and **G** for somatic cell score (Figure 5D) was only marginal.

**Figure 6:**
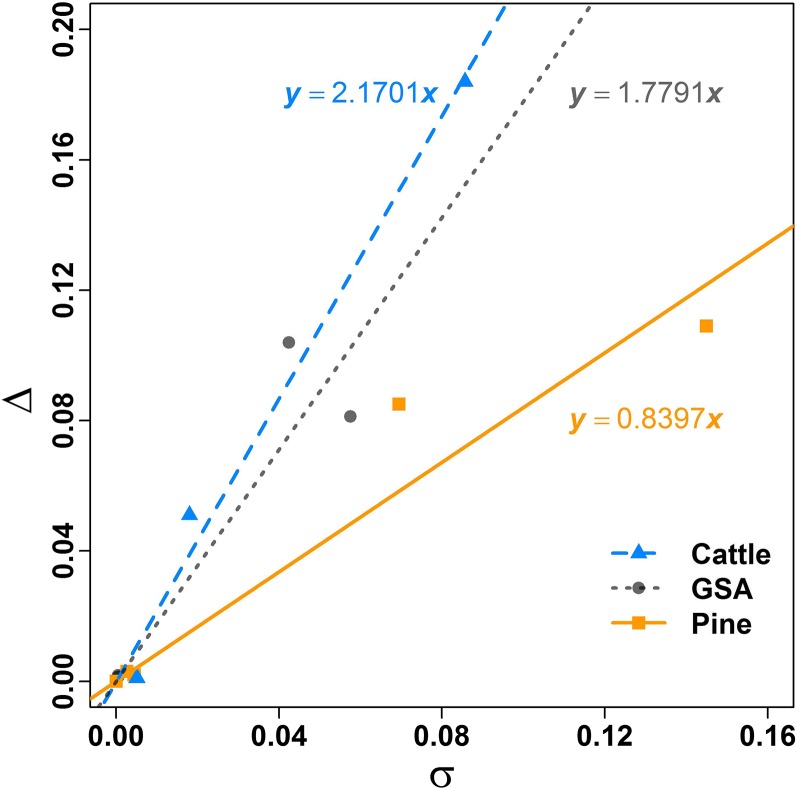
Regression of absolute increased accuracy of best linear unbiased prediction -given genetic architecture (BLUP|GA) over genomic best linear unbiased prediction (∆) on the distance between T and G matrices (σ).

The results suggest that the advantage of BLUP|GA over GBLUP is the more pronounced the more the **T** and **G** matrices differ from each other. Therefore, we quantified the distance between **T** and **G** by calculating the standard deviation of the element-wise difference between the two matrices, *i.e.*, $\sigma =\sqrt{var(T_{ij}-G_{ij})}$. Next we calculated the linear regression of absolute increased accuracy of BLUP|GA over GBLUP (termed ∆ in the following) on σ. The scatter plots and regression lines for each dataset are shown in Figure 6. The intercepts were set to zero for all regressions since zero is the expectation when **T** = **G**. All regression coefficients are significant (*P* < 0.01). The largest regression coefficient was observed for the cattle dataset (2.17) while the smallest one was observed for the pine dataset (0.84).

## Discussion

In the present study, we propose a new strategy of WGP to implement the BLUP|GA approach and validate this strategy in a variety of genetic architectures, including livestock, crop, and simulated datasets. Although many methods exist in this discipline, the new strategy of BLUP|GA provides a plausible alternative to established methods and is shown to flexibly adapt to the given genetic architecture of the trait of interest.

BLUP|GA could effectively improve the predictive performance of WGP compared to GBLUP. We validated the performance of BLUP|GA and GBLUP in four different datasets (Table 1). The validated traits included various genetic architectures, such as traits with major gene(s) [fat percentage in dairy cattle, rust resistant in loblolly pine (Wilcox *et al.* 1996)], traits with loci of moderate effect (milk yield in dairy cattle, three traits in QTL-MAS, GammaUnres and GammaRes in GSA), and traits with only small effect loci (somatic cell score, PolyUnres and PolyRes in GSA). Results showed that BLUP|GA outperformed GBLUP in 20 of 21 scenarios (three traits by seven population sizes) in the dairy cattle dataset (Table 2) and in 12 of 13 traits in the three public datasets (Table 4). In addition, BLUP|GA performed better than BayesA, BayesB, and BayesC in 12 of 13 traits in the three public datasets (Table 5). In the cattle dataset, BLUP|GA showed advantage over BayesB with N = 125 and 500, but not for N = 2000 for the trait fat% (Table S1). Because GBLUP and BayesB were suggested as standard approaches for method validation, our results strongly indicate that BLUP|GA is a promising approach that has the potential to flexibly account for effects of genetic architecture when they are relevant.

An interesting question raised by the favorable performance of BLUP|GA in these tested datasets is, why could BLUP|GA improve the prediction ability compared with GBLUP? The difference between GBLUP and BLUP|GA lies in the variance-covariance matrix. BLUP|GA replaces the **G** matrix with a **T** matrix. Since the **T** matrix was a combination of genetic architecture part (**S** matrix) and the **G** matrix according to equation 4, the favorable performance of BLUP|GA is mainly due to the genetic architecture part (**S** matrix) and the way we build the **T** matrix (weighted sum, equation 4).

To understand the reason of increase in accuracy, we first investigated heat maps of these relationship matrices. The contrast between the heat maps of **S** from three dairy cattle model traits and the **G** matrix (Figure 5), together with the accuracy from GBLUP and BLUP|GA (Table 2), strongly indicate that the genetic architecture part (**S** matrix) could effectively improve the prediction ability of the **G** matrix. The block structure of **S** for FP mainly reflects the three DGAT1 genotypes, which is a validated major gene affecting milk traits in dairy cattle. This finding suggests that the increase in accuracy is possibly due to the increased similarity between **T** and the genetic relationship at unobserved causal loci (de los Campos *et al.* 2013b) compared with **G** matrix used in GBLUP. The improvement of accuracy for SCS is the least, while the similarity between the **S** matrix of SCS and **G** matrix is the greatest among the three traits (Table 2 and Figure 5). In addition, we calculated the regression of increased accuracy of BLUP|GA over GBLUP (∆) on the distance between **T** and **G** matrices (σ). Significant regression coefficients between them indicate that the **T** matrix used in BLUP|GA is the main explanation for the increased accuracy, and the amount of similarity between **S** and **G** matrices also affects the increment of accuracy. These observations together explain why we benefit from the **S** matrix for MY and FP but not for SCS. 

On the basis of these observations, we speculate that for a large number of quantitative traits, the **S** matrix built from their inferred genetic architecture is similar to the averaged **G** matrix. This similarity might come from a (quasi) infinitesimal genetic background or might also arise for a trait that is controlled by a limited but not extremely small number of genes distributed across the genome. If this is the case, it is not hard to image why the **G** matrix is so robust in its prediction ability and why we can successfully apply the same **G** matrix or numerator relationship matrix (**A** matrix (Henderson 1975b)) to genetic evaluation for different traits. Although these approached are robust, they are not optimal for all genetic architectures. With the availability of more precise genetic data (such as whole-genome sequences) and the ongoing research toward a more comprehensive understanding of the underlying genetic architectures, more and more quantitative traits could benefit from more informative **S** matrices built on such external information sources by using the approach suggested in an earlier study (Zhang *et al.* 2014).

The way we build the **T** matrix ensures BLUP|GA being flexible to shift between the infinitesimal and major gene genetic architectures. The **T** matrix comes from a weighted sum of a realized / averaged information matrix (**G**) and a trait specific genetic architecture matrix (**S**) (defined in equation 4). Hence, the selection of the optimal overall weight in equation 4 is important for the performance of BLUP|GA. Previous studies showed that improper weights can lead to suboptimal model predicting ability (Liu *et al.* 2011; Gao *et al.* 2012; Zhang *et al.* 2014). The ideal situation is that the selected weight for **S** is (1) small for infinitesimal model traits to shift the genetic model to be more similar to GBLUP, and (2) large for traits affected by major gene(s) to shift the genetic model to be more similar to BayesB. Expectedly, the actual selected optimal weights are relatively large for the traits fat percentage in cattle and rust resistance in pine, and small for the traits somatic cell score in cattle and PolyUnres in the GSA dataset (Table 3 and Table 4). Hence, given a proper weight and an informative **S** matrix to form a **T** matrix, the predictive ability of BLUP|GA is promising by including this **T** matrix.

To explore the potential of BLUP|GA, three optimal parameters must be provided or fitted: (1) the proportion of top SNPs (*top%*), (2) the number of flanking SNPs adjacent each top SNP (*nflank*), and (3) the overall weight for matrix **S** (*weight*). The three parameters are determined to maximize the predicting ability of BLUP|GA by maximizing the similarity of **T** and the genetic relationship at unobserved causal loci. The first two parameters are used to select the subset of ‘important markers’ while building an **S** matrix. The parameter *nflank* is relevant to the global and local level and range of linkage disequilibrium in the dataset. Although *nflank* is defined as a parameter to capture the linkage disequilibrium among the top SNPs and their flanking SNPs, the contribution of linkage disequilibrium to additive variance is not considered in this approach (Gianola *et al.* 2009). We did not optimize *nflank* for the pine dataset due to the lack of marker map information (*nflank* = 0 for pine, Table 4). In addition, the third parameter and a vector of marker weights corresponding to each selected marker are also used to build the **T** matrix via equation 4 and equation 5.

In this study, we determined the optimal parameters, especially the *top%* and *weight*, through a grid search within the parameter spaces in the training stage. This validation procedure can maximize the predicting ability of BLUP|GA within the reference set, though it may provide only suboptimal parameters for the application stage (Figure 3). Hence, there is room to further improve the performance of WGP, and other means of determining the optimal parameters deserve further investigation. Compared with other variable selection models, such as BayesB, the computational burden of BLUP|GA is low (minutes *vs.* hours), and mainly dependent on the number of genotyped individuals and the training strategy used. The strategy proposed in this study to determine the optimal parameters for BLUP|GA might not be the most efficient one but is at least an effective way to obtain parameters that improve predictive ability, and similar strategies were used in other studies (Liu *et al.* 2011; Gao *et al.* 2012; Zhang *et al.* 2014). Some alternative strategies to choose the BLUP|GA parameters, such as determining weights by variance component estimation, were tested but generally did not lead to improved performance, especially so with reference datasets of limited size (results not shown). More efficient ways to optimize the necessary parameters for BLUP|GA will be investigated in our further studies.

Although results from training stage strongly demonstrate the advantage of BLUP|GA over GBLUP, results from both training stage and application stage are reported for the dairy cattle dataset and the QTLMAS dataset to show the entire computational strategy proposed for BLUP|GA. In practice, a training stage is necessary for BLUP|GA to determine the optimal parameters, especially for *weight* and *top%*. The way to determine the parameters can be very flexible, such as by CV or other means. These optimized parameters can then be used as basis for genomic prediction with BLUP|GA in a candidate population. Though the deviation in accuracies observed between the two stages is different in the two tested datasets (Table 2, Table 3, and Table 4), and the optimal parameters obtained from training stage might not be the best in application stage (Figure 3, 0.838 *vs.* 0.848), the advantage of BLUP|GA over GBLUP exists (Table 2, Table 3, Table 4, and Table 5) in both stages (training stage and application stage). This result indicated that BLUP|GA may be useful also under practical circumstances.

In most implementations of WGP, the GBLUP approach is recommended as a benchmarking method because of its proven robustness. Are there suitable diagnostics that can be derived from the data at hand, which indicate whether the performance of GBLUP can be improved upon by alternative models that take the genomic architecture into account? We suggest as diagnostics the plot of cumulative genetic variance curves (Figures 2 and Figure S3, Figure S4, and Figure S5 for the full and decreased population sizes in the cattle dataset and Figure S6 and Figure S7 for the traits rust_bin and rootnum_bin in the pine dataset). From these curves, it becomes evident that a high proportion of variance explained by a small proportion of top SNPs is indicative for the potential to outperform GBLUP by BLUP|GA or some other approach accounting for genetic architecture. A second diagnostic is the difference between the **G** and the **S** matrix, which can be expected visually (Figure 4 for the three cattle traits) or summarized in a suitable statistic such as the standard deviation of element wise differences σ. Our results suggest that the advantage of BLUP|GA will be the larger, the more differentiated these matrices are. The suggested diagnostics can be derived from a dataset at hand, so that decisions on the choice of an appropriate model can be made on the spot. However, it should be noted that BLUP|GA as implemented in this study is a general approach which encloses GBLUP as a special case (with either *weight* or *top%* being 0), and thus will “automatically” find the best model—which might be close to GBLUP in many cases—when calibrated. So, applying BLUP|GA would ease the burden of model selection in the practice of WGP.

With the rapid reduction of sequencing cost, sequence data will soon be available for the use in breeding programs. In this case, if the number of individuals involved was less than the number of markers, WGP approaches based on some sort of genomic relationship matrix are computationally much more efficient than approaches based on the estimation of SNP effects (Ober *et al.* 2012). On the basis of the aforementioned ideas, it might be an efficient strategy to construct the realized relationship matrix (**G**) with a sample of evenly spaced markers first. Then, one could improve the prediction model of BLUP|GA by building an **S** matrix with the top SNPs extracted from the dataset in hand according to their effect size as proposed in this study, the significance of association (de los Campos *et al.* 2013b), or from public sources as proposed in Kadarmideen (2014) and in our former study (Zhang *et al.* 2014).

The new strategy applied for BLUP|GA is a favorable alternative to the standard GBLUP model, which better accounts for the genetic architecture of the quantitative trait under consideration. This feature is mainly due to the increased similarity between trait specific variance-covariance relationship matrix **T** and the genetic relationship matrix at unobserved causal loci. Given a subset of important markers that possibly locate in QTL regions and proper corresponding weights, genomic prediction could be successfully conducted with the BLUP|GA model in the four datasets analyzed in the present study and in most cases was found to be among the best methods for genomic prediction. The pattern of curves from cumulative proportion of genetic variance explained by top SNPs and the element wise standard deviation between **G** and **T** matrices might be a good diagnostic indicator for model selection.

## 

## Supplementary Material

Supporting Information
