# Supplementary material for: Accuracy of Whole-Genome Prediction Using a Genetic Architecture-Enhanced Variance-Covariance Matrix
Source: G3 (Bethesda). 2015 Feb 9;5(4):615–27. doi: 10.1534/g3.114.016261 (PMC4390577; doi:10.1534/g3.114.016261)
Supplement: Supporting Information [file supp_5_4_615__index.html]

Accuracy of Whole-Genome Prediction Using a Genetic Architecture-Enhanced Variance-Covariance Matrix — Supporting Information 

# Accuracy of Whole-Genome Prediction Using a Genetic Architecture-Enhanced Variance-Covariance Matrix

## Supporting Information for Zhang *et al.*, 2015

**Files in this Data Supplement:**

- Supporting Information - Figures S1-S7, Table S1, and Files S1-S2 (PDF, 637 KB)
- Figure S1 - Manhattan plot of the marker effects estimated for milk yield. (PDF, 206 KB)
- Figure S2 - Manhattan plot of the marker effects estimated for somatic cell score. (PDF, 242 KB)
- Figure S3 - Cumulative proportion of genetic variance explained by SNPs for fat percentage. (PDF, 165 KB)
- Figure S4 - Cumulative proportion of genetic variance explained by SNPs for milk yield. (PDF, 163 KB)
- Figure S5 - Cumulative proportion of genetic variance explained by SNPs for somatic cell score. (PDF, 159 KB)
- Figure S6 - Cumulative proportion of genetic variance explained by SNPs for Rustbin in Loblolly pine dataset. (PDF, 157 KB)
- Figure S7 - Cumulative proportion of genetic variance explained by SNPs for Rootnum\_bin in Loblolly pine dataset. (PDF, 156 KB)
- Table S1 - Performance of BayesB, BLUP|GA, and GBLUP for fat%. (PDF, 127 KB)
- File S1 - Genotypes of German Holstein cattle. This file includes 42,551 SNP genotypes for each of the 5,024 animals. The first column includes animal IDs. The SNP genotypes were 0, 1, 2 for homozygous, heterozygous, and the alternative homozygous, respectively. (.zip, 60 MB)
- File S2 - Phenotypes of German Holstein cattle. This file includes the three phenotype values (conventional EBV) for each of the 5,024 animals. All the EBVs were standardized to mean = 0 and variance = 1. (.txt, 181 KB)
