# Supplementary material for: Accuracy of Whole-Genome Prediction Using a Genetic Architecture-Enhanced Variance-Covariance Matrix
Source: G3 (Bethesda). 2015 Feb 9;5(4):615–27. doi: 10.1534/g3.114.016261 (PMC4390577; doi:10.1534/g3.114.016261)
Supplement: Supporting Information [file supp_g3.114.016261_016261SI.pdf]

G3 · Genes | Genomes | Genetics

# Accuracy of whole genome prediction using a genetic architecture enhanced variance-covariance matrix

**Zhe Zhang[*,§], Malena Erbe[§], Jinlong He[*], Ulrike Ober[§], Ning Gao[*], Hao Zhang[*], Henner Simianer[§,1], Jiaqi Li[*,1]**

[*] **National Engineering Research Center for Breeding Swine Industry, Guangdong Provincial Key Lab of Agro-animal Genomics and Molecular Breeding, College of Animal Science, South China Agricultural University, Guangzhou 510642, China**

[§] **Department of Animal Sciences, Animal Breeding and Genetics Group, Georg-August-Universität Göttingen, Göttingen 37075, Germany**

[1]Corresponding authors:

Henner Simianer

E-mail: hsimian@gwdg.de

Jiaqi Li

E-mail: jqli@scau.edu.cn

**DOI: 10.1534/g3.114.016261**

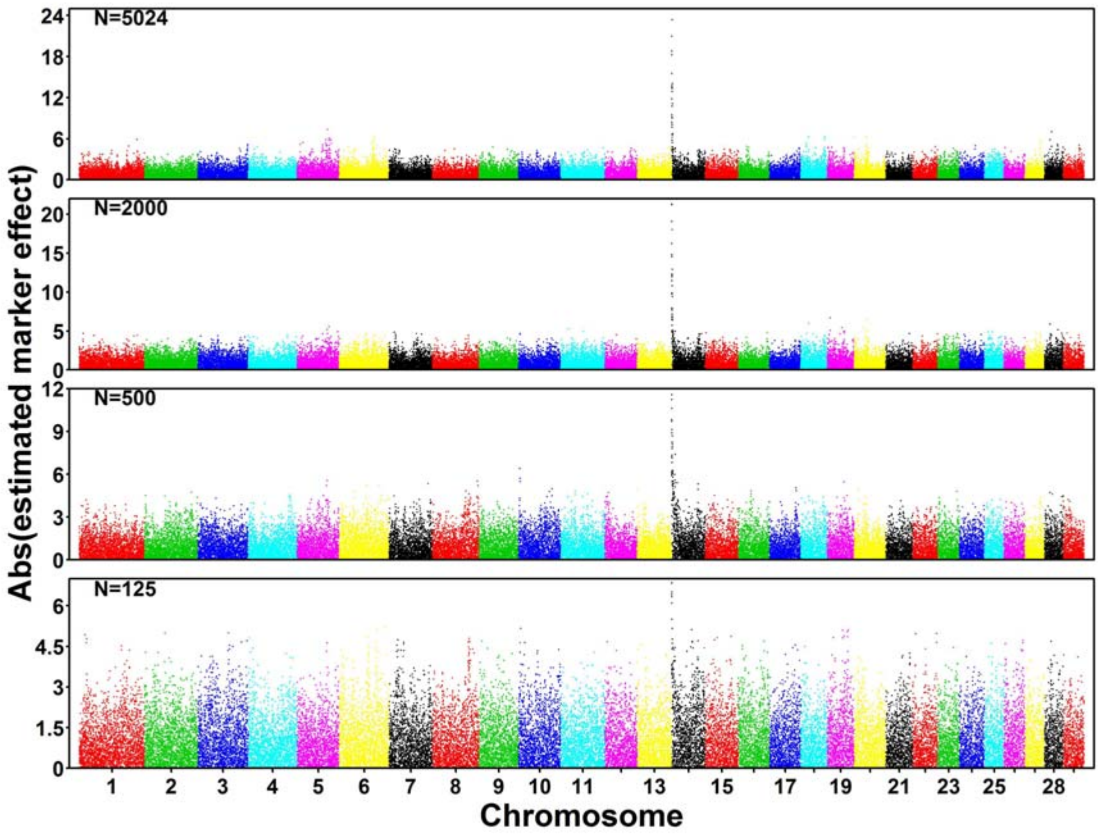


**Figure S1 Manhattan plot of the marker effects estimated for milk yield.** Marker effects ($g_i$) were estimated using RRBLUP and rescaled so that the average marker effect is 1, in order to make the sizes of marker effects from different population sizes (N) or different traits comparable.

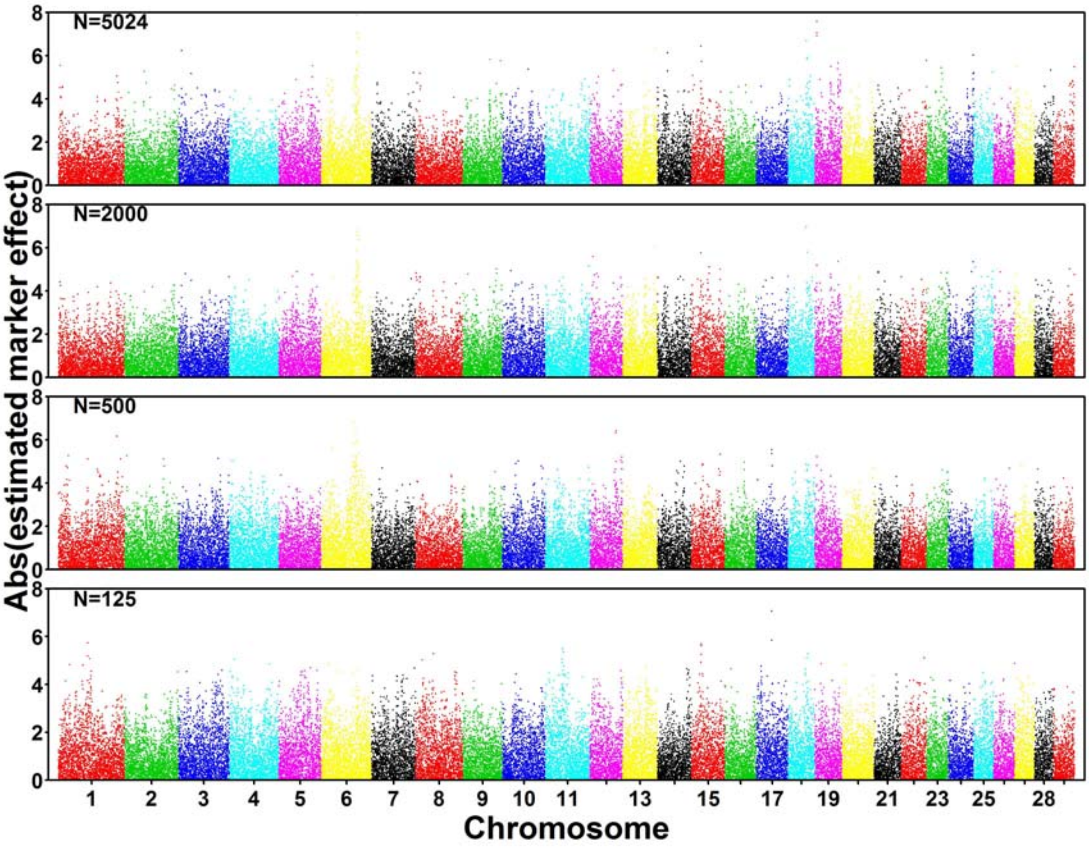


**Figure S2 Manhattan plot of the marker effects estimated for somatic cell score.** Marker effects ($g_i$) were estimated using RRBLUP and rescaled so that the average marker effect was 1, in order to make the sizes of marker effects from different population sizes (N) or different traits comparable.

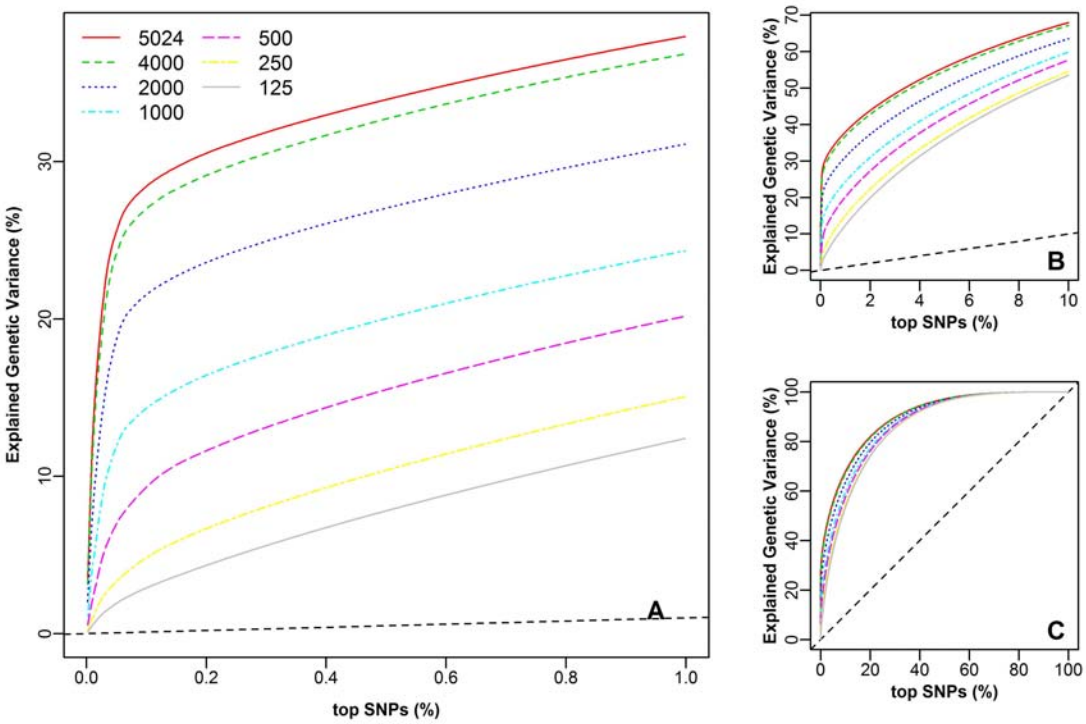


**Figure S3** Cumulative proportion of genetic variance explained by SNPs for fat percentage.

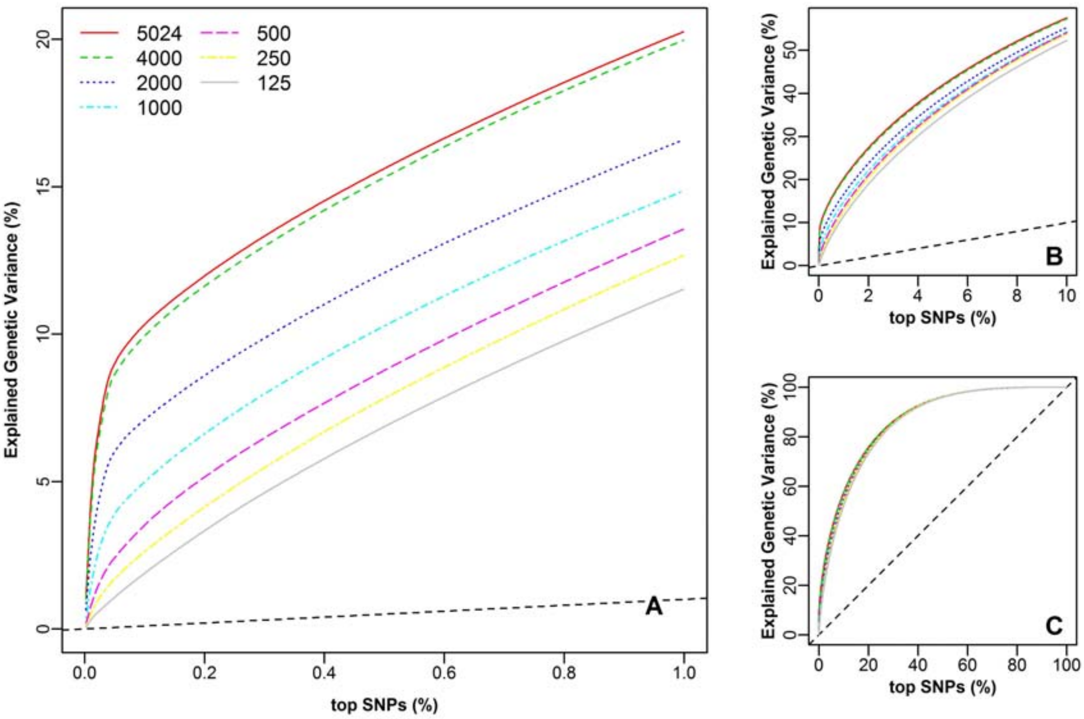


**Figure S4** Cumulative proportion of genetic variance explained by SNPs for milk yield.

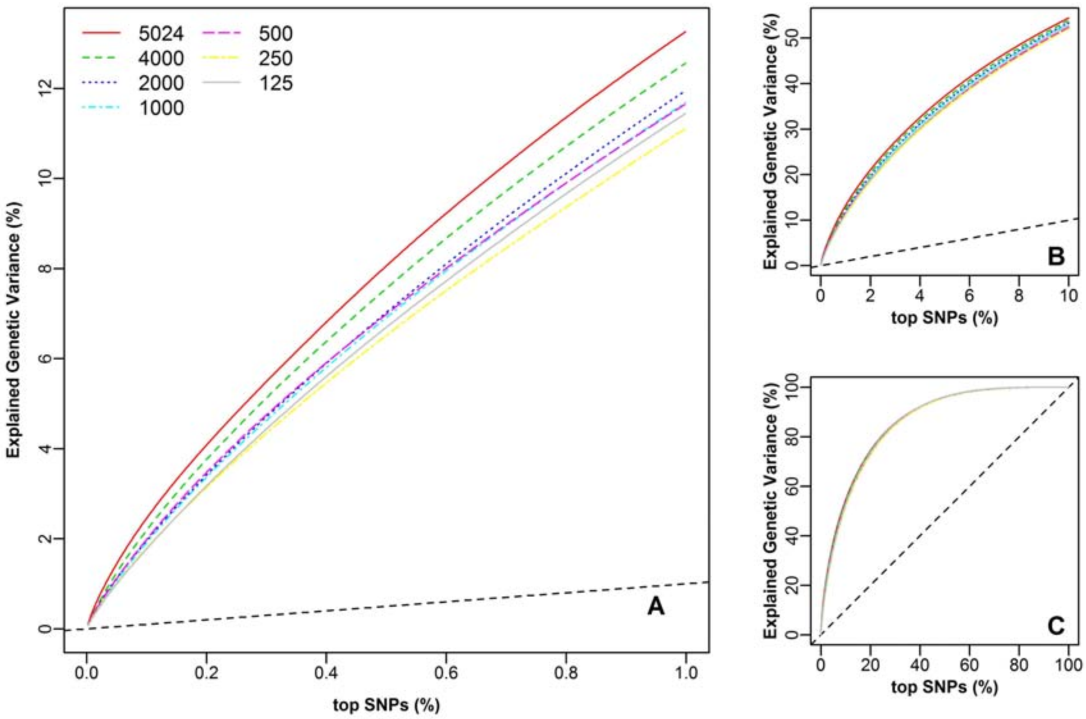


**Figure S5** Cumulative proportion of genetic variance explained by SNPs for somatic cell score.

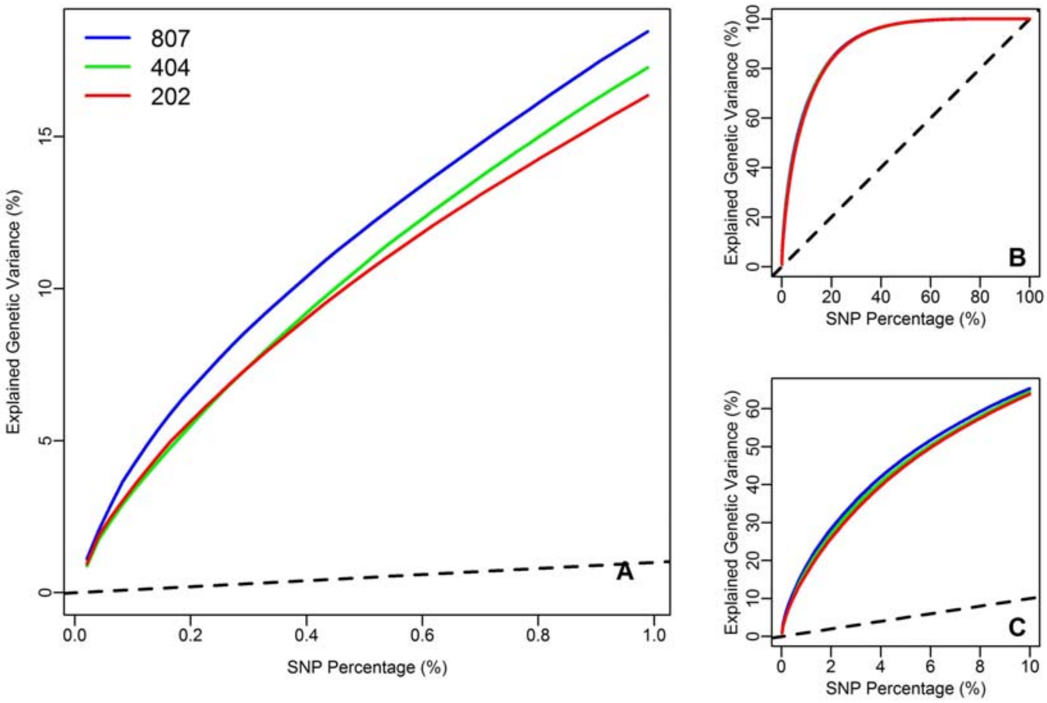


**Figure S6** Cumulative proportion of genetic variance explained by SNPs for Rustbin in Loblolly pine dataset.

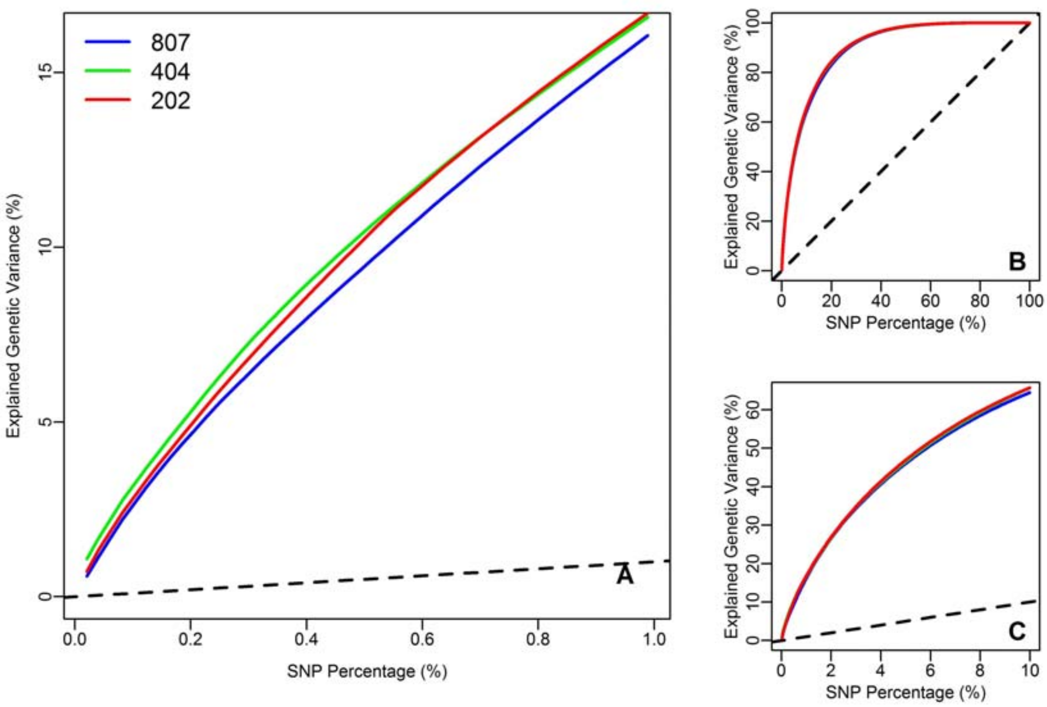


**Figure S7** Cumulative proportion of genetic variance explained by SNPs for Rootnum_bin in Loblolly pine dataset.

**Table S1 Performance of BayesB, BLUP|GA, and GBLUP for fat%**

| *N* | GBLUP | | BLUP\|GA | | BayesB | |
|---|---|---|---|---|---|---|
| | $r_{(EBV, GEBV)}$ | $b_{(EBV,GEBV)}$ | $r_{(EBV, GEBV)}$ | $b_{(EBV,GEBV)}$ | $r_{(EBV, GEBV)}$ | $b_{(EBV,GEBV)}$ |
| 2000 | 0.698±0.001 | 0.997±0.002 | 0.808±0.001 | 0.963±0.002 | **0.813**±0.001 | 0.991±0.002 |
| 500 | 0.557±0.004 | 1.102±0.008 | **0.761**±0.002 | 0.983±0.003 | 0.750±0.002 | 0.997±0.004 |
| 125 | 0.371±0.010 | 1.108±0.032 | **0.676**±0.005 | 0.959±0.011 | 0.652±0.007 | 1.077±0.017 |

**Files S1-S2**

**Available for download at http://www.g3journal.org/lookup/suppl/doi:10.1534/g3.114.016261/-/DC1**

**File S1 Genotypes of German Holstein cattle**

This file includes 42,551 SNP genotypes for each of the 5,024 animals. The first column includes animal IDs. The SNP genotypes were 0, 1, 2 for homozygous, heterozygous, and the alternative homozygous, respectively.

**File S2 Phenotypes of German Holstein cattle**

This file includes the three phenotype values (conventional EBV) for each of the 5,024 animals. All the EBVs were standardized to mean = 0 and variance = 1.
