# Supplementary material for: Accuracy of Whole-Genome Prediction Using a Genetic Architecture-Enhanced Variance-Covariance Matrix
Source: G3 (Bethesda). 2015 Feb 9;5(4):615–27. doi: 10.1534/g3.114.016261 (PMC4390577; doi:10.1534/g3.114.016261)
Supplement: Supporting Information [file supp_g3.114.016261_FigureS2.pdf]

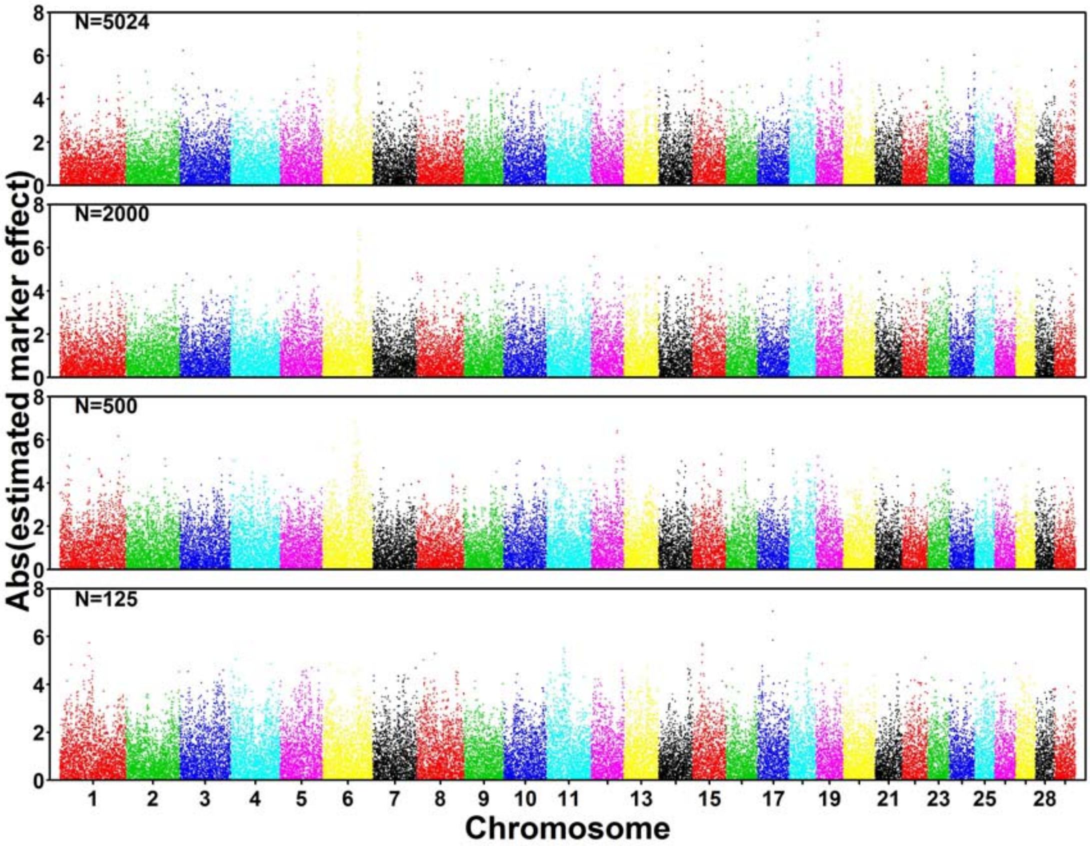

Figure S2 Manhattan plot of the marker effects estimated for somatic cell score. Marker effects (g_i) were estimated using RRBLUP and rescaled so that the average marker effect was 1, in order to make the sizes of marker effects from different population sizes (N) or different traits comparable.
