# Supplementary figures and images for: Accuracy of Whole-Genome Prediction Using a Genetic Architecture-Enhanced Variance-Covariance Matrix
Source: G3 (Bethesda). 2015 Feb 9;5(4):615–27. doi: 10.1534/g3.114.016261 (PMC4390577; doi:10.1534/g3.114.016261)

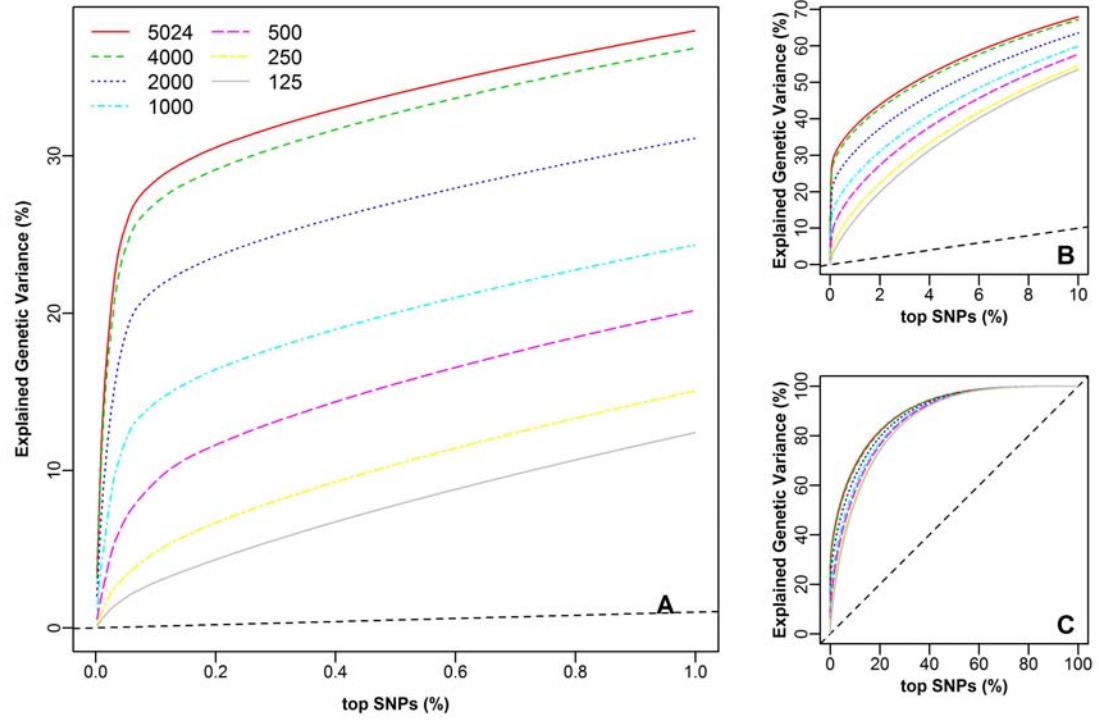

**Figure S3** Cumulative proportion of genetic variance explained by SNPs for fat percentage.

Supplement: Supporting Information [file supp_g3.114.016261_FigureS3.pdf]

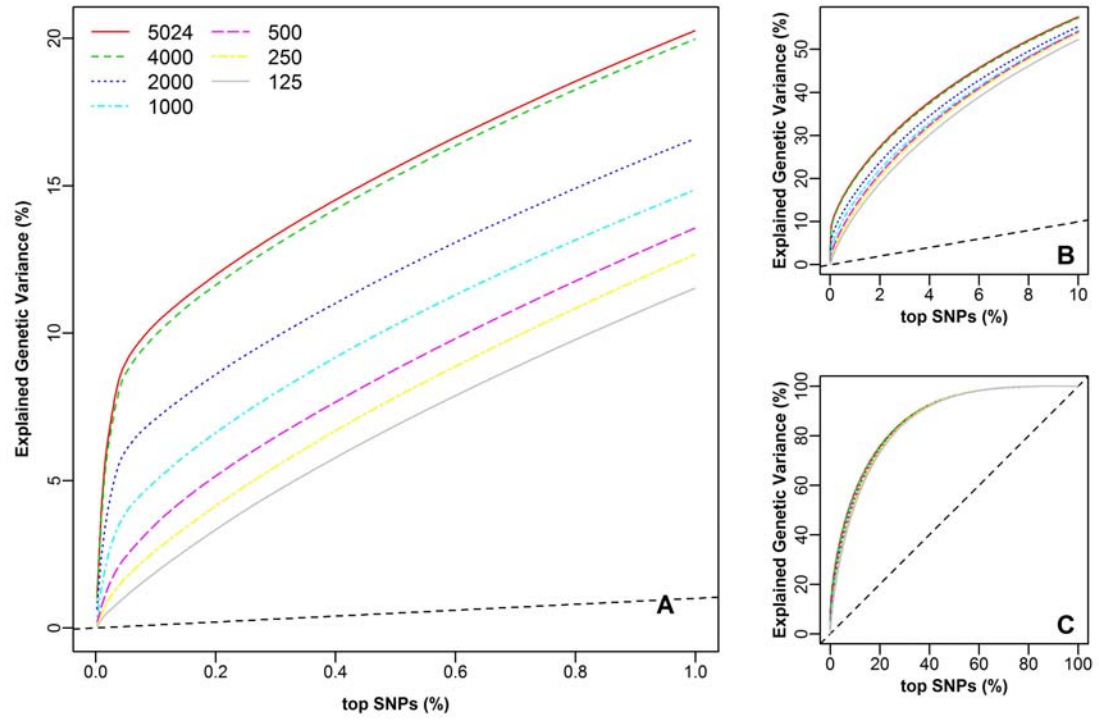

**Figure S4** Cumulative proportion of genetic variance explained by SNPs for milk yield.

Supplement: Supporting Information [file supp_g3.114.016261_FigureS4.pdf]

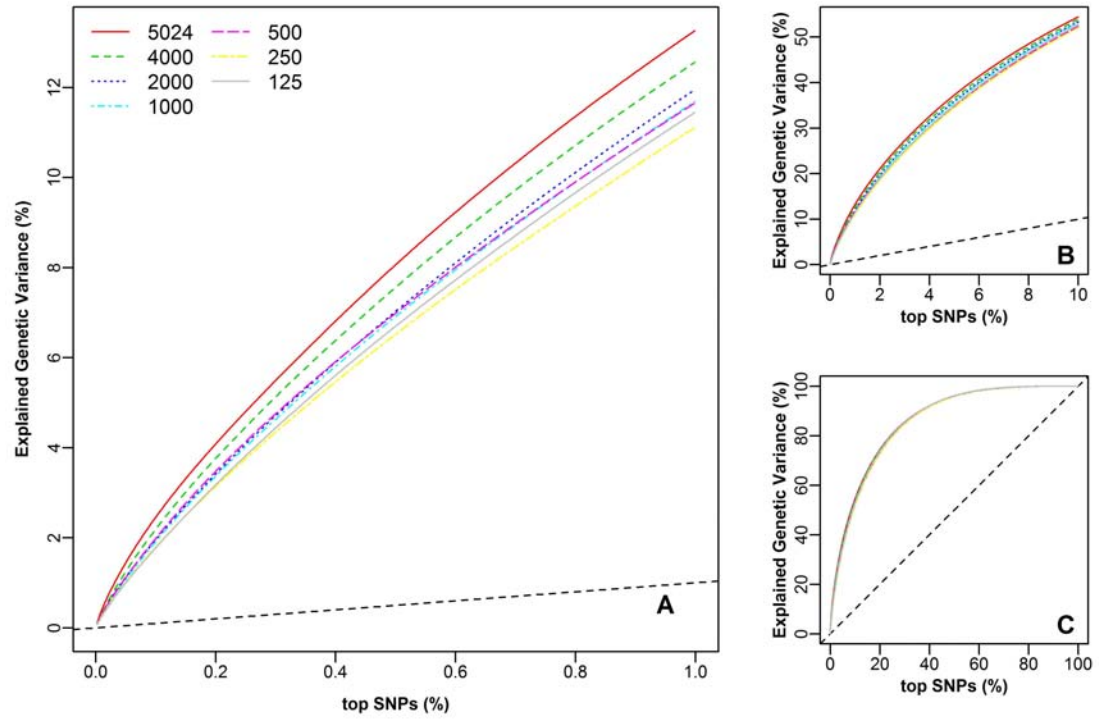

**Figure S5** Cumulative proportion of genetic variance explained by SNPs for somatic cell score.

Supplement: Supporting Information [file supp_g3.114.016261_FigureS5.pdf]
