# Supplementary material for: Accuracy of Whole-Genome Prediction Using a Genetic Architecture-Enhanced Variance-Covariance Matrix
Source: G3 (Bethesda). 2015 Feb 9;5(4):615–27. doi: 10.1534/g3.114.016261 (PMC4390577; doi:10.1534/g3.114.016261)
Supplement: Supporting Information [file supp_g3.114.016261_FigureS7.pdf]

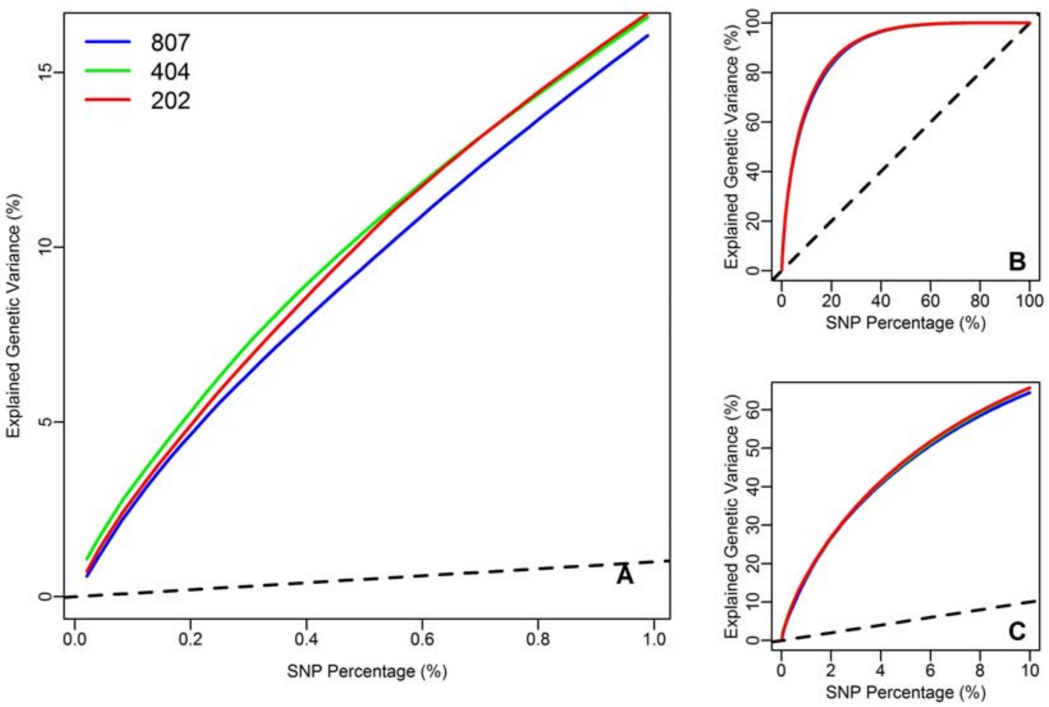


**Figure S7** Cumulative proportion of genetic variance explained by SNPs for Rootnum_bin in Loblolly pine dataset.
