# Supplementary material for: Accuracy of Whole-Genome Prediction Using a Genetic Architecture-Enhanced Variance-Covariance Matrix
Source: G3 (Bethesda). 2015 Feb 9;5(4):615–27. doi: 10.1534/g3.114.016261 (PMC4390577; doi:10.1534/g3.114.016261)
Supplement: Supporting Information [file supp_g3.114.016261_TableS1.pdf]

Table S1 Performance of BayesB, BLUP|GA, and GBLUP for fat%

N	GBLUP		BLUP|GA		BayesB	
	$r_{(\mathrm{EBV,\ GEBV})}$	$b_{(\mathrm{EBV,GEBV})}$	$r_{(\mathrm{EBV,\ GEBV})}$	$b_{(\mathrm{EBV,GEBV})}$	$r_{(\mathrm{EBV,\ GEBV})}$	$b_{(\mathrm{EBV,GEBV})}$
2000	0.698±0.001	0.997±0.002	0.808±0.001	0.963±0.002	**0.813**±0.001	0.991±0.002
500	0.557±0.004	1.102±0.008	**0.761**±0.002	0.983±0.003	0.750±0.002	0.997±0.004
125	0.371±0.010	1.108±0.032	**0.676**±0.005	0.959±0.011	0.652±0.007	1.077±0.017
